# Deciphering the role of RNA in regulating CTCF’s DNA binding affinity in leukemia cells

**DOI:** 10.1186/s13059-025-03582-x

**Published:** 2025-05-12

**Authors:** Judith Hyle, Wenjie Qi, Mohamed Nadhir Djekidel, Wojciech Rosikiewicz, Beisi Xu, Chunliang Li

**Affiliations:** 1 Department of Tumor Cell Biology, St. Jude Children’s Research Hospital, 262 Danny Thomas Place, Memphis, TN 38105 USA; 2 Center for Applied Bioinformatics, St. Jude Children’s Research Hospital, 262 Danny Thomas Place, Memphis, TN 38105 USA

**Keywords:** CTCF, RNA, Transcription, Auxin-inducible degron, Chromatin accessibility

## Abstract

**Background:**

CTCF, a highly studied transcription factor, is essential for chromatin interaction maintenance. Several independent studies report that CTCF interacts with RNAs in vitro and in cells. Yet continuous debates about the authenticity of the RNA-binding affinity of CTCF and its biological role remain in large part due to limited research techniques available, such as CLIP-seq.

**Result:**

Here, we investigate RNA’s role in CTCF’s transcription factor function through its chromatin occupancy. To systematically explore whether RNAs affect CTCF’s ability to bind DNA, we perturb CTCF-RNA interactions by three independent approaches and examine CTCF genome occupancy by ChIP-seq. Although RNase A and triptolide treatment each affect a certain number of CTCF-binding peaks, few peaks overlap between treatment groups indicating the effect of RNA in regulating CTCF’s DNA binding affinity is variable between loci. In addition, limited transcriptional or chromatin accessibility changes occur between cells expressing wild-type CTCF or CTCF lacking the RNA binding region.

**Conclusion:**

Our data provide a complementary approach and in silico evidence to consider the significance of RNA affecting CTCF’s DNA binding affinity globally.

**Supplementary Information:**

The online version contains supplementary material available at 10.1186/s13059-025-03582-x.

## Background

CTCF is a zinc finger (ZF)-containing transcription factor (TF) that plays essential roles in chromatin looping maintenance, transcription repression and activation, and chromatin accessibility control [1–6]. Biochemistry and molecular biology evidence revealed that CTCF mainly functions through its conserved 11 repeat ZF domains and directly interacts with target DNA carrying consensus CTCF motifs [6–11]. Recently, several independent groups reported that CTCF could bind to RNAs in vitro [12–15], and a CTCF-mediated phase separation model has been proposed [16, 17]. Since transcription factors usually do not harbor classic RNA-binding domains, most studies relied on the CLIP-seq technique to cross-link CTCF and RNAs, followed by immunoprecipitation of target proteins to pull down UV-crosslinked interacting RNAs [12, 15]. For instance, Oksuz et al. claimed that a broad spectrum of TFs, including CTCF, bind RNAs through the arginine-rich RNA-binding motif (ARM) [12]. Saldana-Meyer et al. concluded that CTCF’s ZFs 1 and 10 function through interactions with RNAs by utilizing an auxin-inducible degron (AID) system and inducible overexpression of ZF mutant forms in mouse embryonic stem cells. The RNA-interaction capability of CTCF stabilized chromatin binding and had some effects on gene expression and chromatin organization regulation as well as a modest decrease in CTCF chromatin binding genome-wide [13]. Our study in the acute lymphoblastic leukemia cell line SEM showed deletions to ZF1 and ZF10 resulted in moderate changes to CTCF binding and dysregulated transcription of a subset of genes regulated by CTCF. However, the analysis showed CTCF binding sites that required either ZF harbored a unique upstream or downstream motif in addition to the conserved consensus DNA motif, suggesting the abrogated binding was due to loss of CTCF-DNA contacts [11]. Another study revealed that the RNA binding region (RBR) at the C-terminus of CTCF orchestrated RNA-dependent chromatin organization and loss of this region resulted in a general reduction in CTCF binding [18]. Conversely, others raised concerns about the low stringency biochemistry CLIP-seq conditions required to preserve native protein-RNA interactions [19]. Data analysis concerns for characterizing CTCF-RNA interactions were also raised [20]. Additionally, the observation of CTCF binding to RNAs has mainly been *in trans* further complicating the ability to decipher its accurate in vivo effect on CTCF functions at the DNA level. Therefore, a continuous debate remains about whether CTCF-RNA interactions represent interactions in vivo and contribute to gene regulation. Notably, most of the work was conducted in mouse embryonic stem cells. Therefore, it is important to investigate the role of RNAs on CTCF-DNA binding in a different cell model.

In this study, we evaluated whether RNAs affected CTCF’s functional role as a transcription factor to bind DNA in a human B-cell leukemia model. We reasoned that if the RNA-binding function of CTCF is indeed essential, loss of RNAs or CTCF’s RNA-binding region would reproducibly impair CTCF’s DNA occupancy in vivo, altering transcriptional regulation and chromatin accessibility. To this end, we leveraged our unique CTCF acute depletion and inducible expression system along with cutting-edge molecular profiling techniques to quantify genome-wide changes upon CTCF-RNA interaction interference. Our results suggested that when disrupting RNA interactions with CTCF in vivo, despite certain changes in selective loci, the DNA occupancy of CTCF was not altered at the genome-wide scale, and changes to transcription and open chromatin organization were minimal.

## Results

**Fig. 1:**
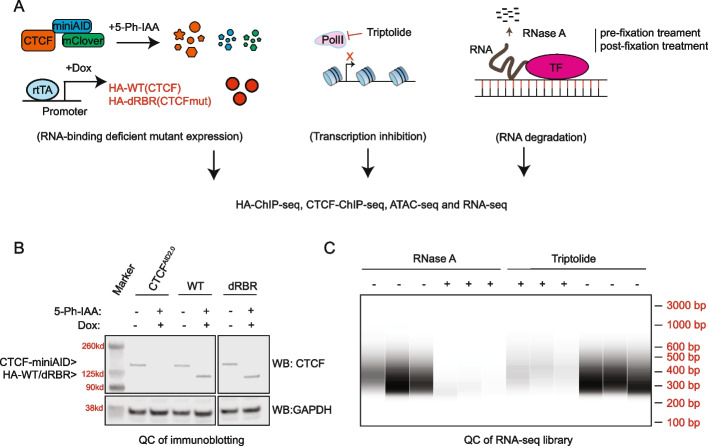
Establish a cellular model to interfere with CTCF-RNA interactions to study the impact on CTCF’s DNA binding affinity. **A** Schematic diagram illustrating three techniques to disrupt CTCF-RNA interactions. On the left is an illustration of how the ectopic HA-tagged CTCF swap system works in combination with acute protein degradation of endogenous CTCF. The homozygous miniAID-mClover3 knockin SEM cell lines CTCF^AID2/WT^ and CTCF^AID2/dRBR^ were previously generated [11]. When added to cell culture, the 5-Ph-IAA auxin analog acts as a ligand to bind to the miniAID tag (fused to endogenous CTCF protein) and OsTIR1(F74G) protein to promote acute protein degradation through ubiquitination by the SCF complex. After 6 h of 5-Ph-IAA treatment, the CTCF HA-tagged WT or CTCF-HA-dRBR ectopic proteins were induced by doxycycline for a total of 18 h of doxycycline and 24 h of 5-Ph-IAA treatment. In the middle is an illustration of transcription inhibition by the natural product, triptolide. Triptolide was added to live cell culture to block the PolII activity and global nascent transcription. On the right is a diagram showing how RNase A was used during the ChIP-seq procedure, either added before (pre-fixation treatment) or after (post-fixation treatment) the chromatin fixation, to degrade global RNAs. **B** Immunoblot analysis of endogenous (CTCF^AID2.0^) and induced exogenous (HA-CTCF) expression of CTCF using an antibody for CTCF. CTCF^AID2.0^ expression can be seen in all untreated samples (−, −). After 6 h of 10 μM 5-Ph-IAA treatment, CTCF^AID2^ protein expression is degraded. Exogenous expression of HA-tagged CTCF wildtype and dRBR mutant is comparable to endogenous CTCF following 18 h of 1 μg/mL doxycycline with concurrent 10 μM 5-Ph-IAA treatment (+, +). GAPDH was included as a loading control. **C** Quality control of RNA inhibition upon triptolide and RNase A treatment. Total RNAs were collected after drug treatment, followed by reverse transcription. The cDNA was fragmented, amplified, and quantified by a bioanalyzer. Three replicates were included for each treatment

Using a standard chromatin immunoprecipitation and sequencing assay (ChIP-seq), we systematically explored the DNA binding affinity of CTCF in three independent and complementary conditions that were either deficient for global RNAs or the RNA-binding region (RBR) of CTCF. (1) We created a protein swap system by combining our established CTCF auxin-inducible degron system with induced expression of CTCF wild-type (WT) or a mutant that lacked the RNA-binding region (Fig. 1A–B, Additional file 1: Fig. S1 A) [11]. (2) In live cell culture, total RNAs, especially nascent and short half-life RNAs, were inhibited by triptolide, the natural product to inhibit XPB, a subunit of TFIIH [21], which induced proteasome-dependent degradation of RNA polymerase II (Fig. 1A, C) [22, 23]. (3) During the ChIP-seq procedure, RNase A was either added to formaldehyde cross-linked chromatin (post-fixation treatment) or cells prior to formaldehyde cross-linking (pre-fixation treatment) to degrade global RNAs (Fig. 1A, C). We hypothesized that if purported CTCF-RNA interactions were essential for CTCF’s DNA binding affinity, we would observe reproducible differential CTCF-binding peaks overlapped from the three conditions.

For these studies, the B-cell lymphoblastic leukemia cell line SEM with constitutive expression of OsTIR1(F74G), endogenous CTCF-miniAID-mClover (CTCF^AID2^), and inducible exogenous HA-tagged wild-type CTCF (CTCF^AID2/WT^) or RBR deficient CTCF (CTCF^AID2/dRBR^) were used [11]. Upon the addition of 5-Ph-IAA, the drug ligand binds specifically to OsTIR1(F74G) to direct the Skp, Cullin, F-box (SCF) complex to AID-tagged CTCF-fusion proteins for ubiquitination and degradation (Additional file 1: Fig. S1 A) [24]. This unique cellular model allowed a quick switch from endogenous CTCF to the ectopic HA-tagged CTCF-WT and dRBR-mutant forms at a similar expression level (Fig. 1B, Additional file 1: Fig. S1B). These ectopically expressed forms of CTCF tagged with HA were immunoprecipitated by anti-HA beads across the following ChIP-seq assays to keep the procedure consistent and comparable and to exclude the residual, undegraded endogenous CTCF, which might interfere with direct comparisons between CTCF-WT and CTCF-dRBR. Treatment groups compared in the HA-ChIP study were (1) triptolide vs DMSO, (2) RNase A vs DMSO, (3) CTCF-dRBR vs CTCF-WT, and (4) and all groups together.

**Fig. 2:**
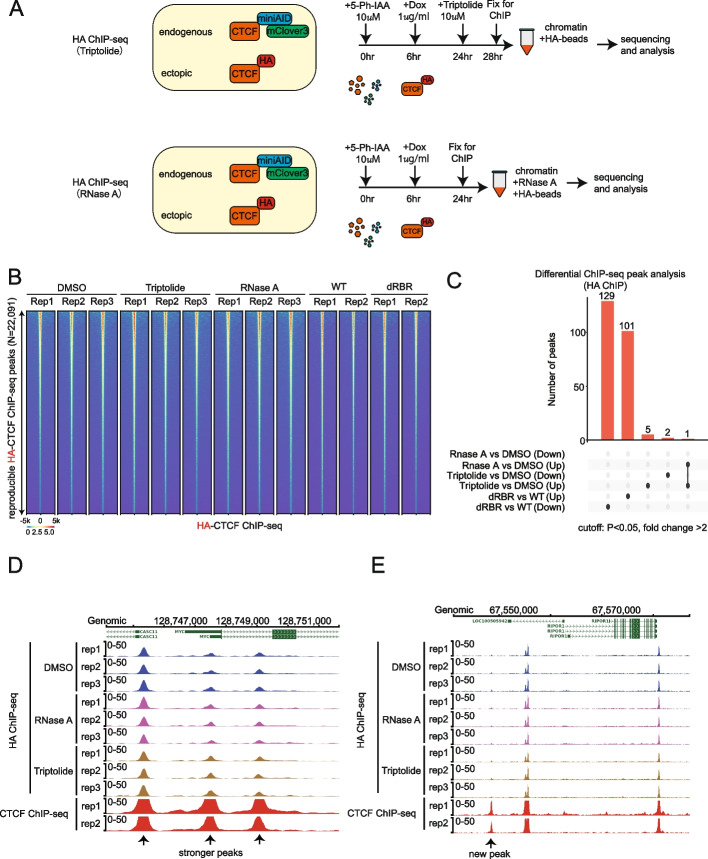
Evaluate CTCF’s DNA binding affinity by HA-ChIP-seq against ectopically expressed HA-tagged CTCF in CTCF RNA binding region deficient cells and cells depleted for global RNAs. **A** Schematic diagram illustrating how the HA-CTCF-ChIP-seq works in combination with triptolide and RNase A treatment. **B** Genomic heatmap of reproducible CTCF peaks from HA-ChIP of HA-tagged CTCF from CTCF^AID2/WT^ cells treated with DMSO, triptolide, and RNase A. HA-CTCF-WT and HA-CTCF-dRBR ChIP-seq tracks were adapted from a previous study (GSE205218). **C** Summary of differential peaks by paired analysis. Up and down peaks were defined by comparing treatment groups vs DMSO, or dRBR vs WT at the cutoff of FC > 2 and *P* value < 0.05. Overlapped peaks were connected by the solid line. **D** ChIP-seq tracks from triptolide, RNase A, DMSO at the *MYC* locus show consistent CTCF binding across all samples (three replicates for each treatment). CTCF ChIP-seq was conducted in the same cells to compare with the HA-ChIP-seq (two replicates for each treatment). **E** ChIP-seq tracks from triptolide, RNase A, DMSO at the *RIPOR1* locus show consistent CTCF binding across all samples. CTCF ChIP-seq was conducted in the same cells to compare with the HA-ChIP-seq (two replicates for each treatment)

For triptolide and RNase A conditions, CTCF^AID2/WT^ cells were treated for 6 h with 5-Ph-IAA to remove endogenous CTCF followed by doxycycline induction of HA-tagged CTCF concurrent with 5-Ph-IAA for a total of 18 h of doxycycline induction and 24 h of 5-Ph-IAA treatment followed by 4 h of either DMSO or triptolide treatment (Fig. 2A). For RNase A HA-ChIP, RNase A was added to chromatin from formaldehyde cross-linked CTCF^AID2/WT^ cells during HA immunoprecipitation (Fig. 2A). Given that both drug treatment approaches focused on whether loss of RNAs would impair CTCF’s ability to bind DNA, we provided a complementary analysis comparing the DNA binding affinity of CTCF-WT and dRBR proteins. A ChIP-seq dataset comparing CTCF^AID2/dRBR^ and CTCF^AID2/WT^ was reanalyzed from our previous study [11].

When peak calling was conducted in each sample against input controls, consistent peak numbers were obtained ranging from 9946 to 25,141. Combined analysis of the HA-ChIP-seq for DMSO, triptolide, RNase A, CTCF-WT, and CTCF-dRBR samples identified 22,091 reproducible CTCF-binding peaks. No dramatic change in peak numbers was observed among the groups (Fig. 2B). The Spearman’s correlation also showed that the ChIP-seq signal pattern clustered closely in all three treatments (Additional file 1: Fig. S1 C). Differential CTCF-binding peak analysis was conducted among paired samples. Up and downregulated peaks were defined by the cutoff as fold change > 2 and *P* value < 0.05. Under this criteria, minimal numbers were found (dRBR vs WT, 230 peaks; RNase A vs DMSO, one peak; triptolide vs DMSO, eight peaks), and there was no significant overlap among the three conditions (Fig. 2C).

**Fig. 3:**
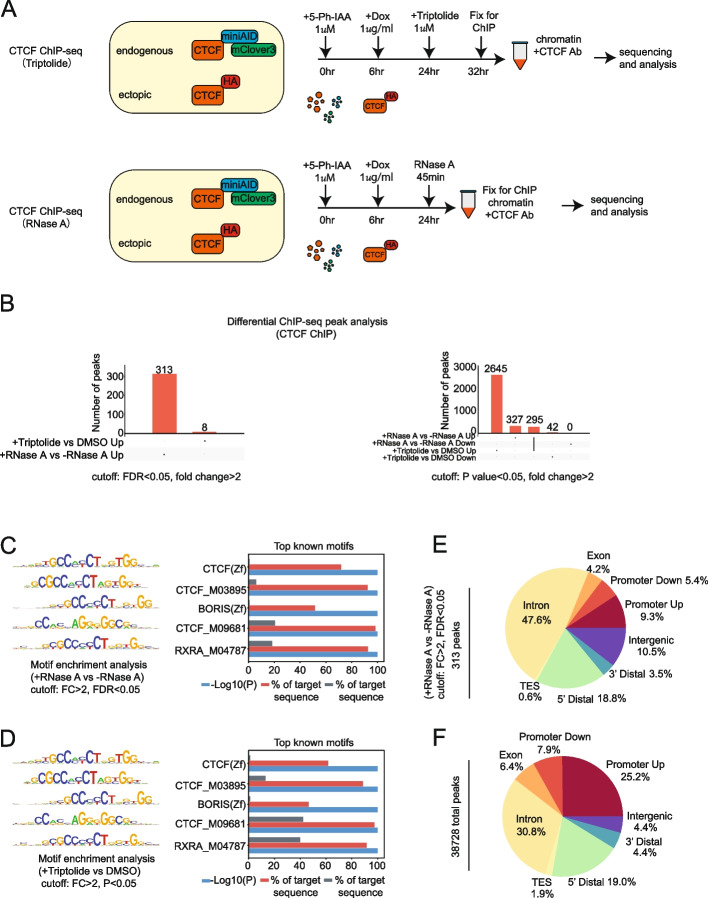
Evaluate the impact of global RNA depletion on CTCF’s DNA binding affinity by CTCF ChIP-seq. **A** Schematic diagram illustrating how the CTCF-ChIP-seq works in combination with triptolide and RNase A treatment. **B** Summary of differential CTCF-binding peaks by paired analysis. Up and down peaks were defined by comparing triptolide vs DMSO and +/− RNase A groups at cutoffs with different stringencies; high stringent cutoff: fold change FC > 2 and FDR < 0.05; modest stringent cutoff: FC > 2 and *P* < 0.05. Overlapped peaks between different comparisons were connected by the solid line. **C** Motif analysis of 313 differential CTCF-binding peaks collected from RNase A treatment vs no treatment. The top 5 were shown according to Homer known motif analysis. **D** Motif analysis of differential CTCF-binding peaks collected from triptolide treatment vs DMSO treatment with the cutoff of FC > 2 and *P* < 0.05. The top 5 were shown according to Homer known motif analysis. **E** Genomic distribution of differential CTCF-binding peaks collected from RNase A treatment vs no treatment. With the cutoff of FC > 2 and FDR < 0.05, about 313 peaks were identified and assigned to different genomic regions. **F** Genomic distribution of total CTCF-binding peaks (38,728)

When comparing the HA-tagged ChIP-seq to CTCF-ChIP-seq peaks, more CTCF peaks and an overall increase in peak signal intensity were observed in the CTCF-ChIP-seq setting (Fig. 2D–E, Additional file 2: Table S1). Statistical comparison of the peak score between the two groups confirmed that HA-ChIP pulled down stronger CTCF binding peaks (Additional file 1: Fig. S1D). Therefore, CTCF antibody-based ChIP-seq was carried out again for triptolide and RNase A treated samples to see if loss of RNA would abrogate CTCF chromatin contacts over a larger population of peaks (Fig. 3A). Triptolide treatment validations were conducted to determine the proper dosage for SEM cells. A time-course and dose-dependent test revealed that a 1-µM treatment for up to 8 h was sufficient to inhibit transcription of CTCF’s target genes *MYC*, *CTCF*, and *RBM45* without inducing significant cell death (Additional file 1: Fig. S2 A–C). For the RNase A ChIP, permeabilized cells were treated with RNase A treatment before chromatin fixation to degrade total RNAs and explore the impact of RNA loss on CTCF binding to DNAs in a naïve condition. RNase A treatment conditions were first validated by incubating permeabilized cells with 1 mg/mL RNase A over a time course. Q-PCR accessed the transcript reduction of *MYC*, a short half-life transcript, and *GAPDH*, a long half-life transcript, as reflected by increasing Ct values. The optimal treatment time of 45 min was chosen (Additional file 1: Fig. S2D). To confirm the global RNA degradation and inhibition in samples prepared for ChIP-seq, fractions of triptolide and RNase A-treated cells were collected for RNA extraction, cDNA reverse transcription, library construction, and quantification. As shown by bioanalyzer measurement, a remarkable reduction of total cDNA levels was achieved compared with no treatment controls (Fig. 1C). Q-PCR also demonstrated a dramatic decrease in mRNA levels for *MYC*, *RBM45*, *CTCF*, and *GAPDH* upon RNase A treatment of ChIP-seq samples (Additional file 1: Fig. S2E). Immunoblotting of fractions from the CTCF-ChIP samples confirmed enrichment of CTCF comparable to input (Additional file 1: Fig. S2 F–G).

For CTCF ChIP-seq, *Drosophila* spike-in controls were added for global normalization. In general, the Spearman’s correlation showed that the ChIP-seq signal patterns clustered closely in both treatments (Additional file 1: Fig. S3 A–B). As expected, more reproducible peaks were called in triptolide/DMSO (69,222) and RNase A treatment (38,728) groups in comparison to the HA-ChIP-seq (22,091) (Additional file 1: Fig. S3 C–D). Less peaks were observed from the RNase A +/− groups likely due to the extended treatment conditions before chromatin fixation. The differential CTCF peaks were called between triptolide and DMSO and +/− RNase A groups separately based on different cutoffs. At the most stringent cutoff (FDR < 0.05 and FC > 2), no downregulated peaks were found in either group. About 313 upregulated peaks were identified from the + RNase A vs − RNase A group and 8 from triptolide vs DMSO. However, none of these upregulated peaks overlapped, and they demonstrated a weaker CTCF binding affinity. When using a lower cutoff of *P* value < 0.05 and FC >2, 2940 upregulated peaks were identified from the triptolide vs DMSO group and 622 from + RNase A vs − RNase A, of which 295 peaks were shared. However, very few downregulated peaks were identified (42 from the triptolide vs DMSO group and zero from + RNase A vs − RNase A) (Fig. 3B). To confirm whether these differential peaks were truly CTCF-binding peaks, the Homer motif analysis was conducted by comparing the differential peaks with background. All top five motifs were matched to different CTCF motifs based on statistical analysis. Also, more than 70% of the 313 upregulated CTCF-binding peaks from the + RNase A vs − RNase A group (FDR < 0.05 and FC > 2) were shown for the CTCF consensus motif (Fig. 3C). Similarly, more than 60% of the 2940 upregulated CTCF-binding peaks from the triptolide vs DMSO group (*P* < 0.05 and FC > 2) contained the CTCF consensus motif (Fig. 3D). The distribution of 313 upregulated peaks from the + RNase A vs − RNase A group (FDR < 0.05 and FC > 2) demonstrated a notable increase of intron-binding peaks and a reduction in promoter-binding patterns compared with the expected distribution (Fig. 3E–F). Collectively, these data suggest that both RNA-depletion strategies affected a small fraction of authentic CTCF-binding peaks. However, the impact of CTCF-RNA interaction in regulating its DNA binding affinity is variable between loci.

**Fig. 4:**
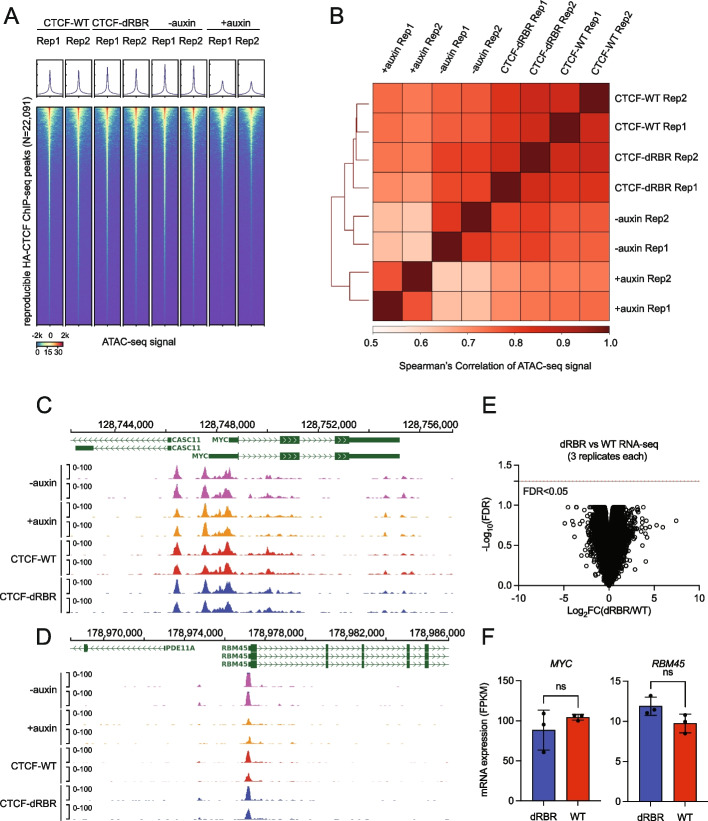
CTCF’s RNA binding domain deficiency or global RNA depletion does not impact genome-wide chromatin accessibility. **A** Genomic heatmap of ATAC-seq signals matched to reproducible CTCF peaks from CTCF^AID2/WT^ cells and CTCF^AID2/dRBR^. ATAC-seq tracks from CTCF^AID2^ with or without auxin (5-Ph-IAA) treatment were shown as controls. **B** Spearman’s correlation of all ATAC-seq signals defined by **A** was calculated to quantify the similarity between samples. **C** ATAC-seq tracks from CTCF-WT and CTCF-dRBR at the *MYC* locus show consistent chromatin accessibility patterns across all samples. CTCF^AID2^ with or without auxin (5-Ph-IAA) treatment were shown as controls. **D** ATAC-seq tracks from CTCF-WT and CTCF-dRBR at the *RBM45* locus show consistent chromatin accessibility patterns across all samples. CTCF^AID2^ with or without auxin (5-Ph-IAA) treatment were shown as controls. **E** Total RNA-seq was performed to quantify the global gene expression changes between CTCF-dRBR and CTCF-WT groups based on the cutoff of FDR < 0.05. *N* = 3. **F** The mRNA expression of CTCF target genes *RBM45* and *MYC* between CTCF-dRBR and CTCF-WT groups. n.s., no statistic difference, calculated by unpaired *t*-test. *N* = 3

We have previously shown that acute depletion of CTCF protein by the AID system rewires approximately 10% of genome-wide chromatin accessibility [2]. To determine the impact of RNA interaction of CTCF in this process, we conducted ATAC-seq in CTCF^AID2/WT^ and CTCF^AID2/dRBR^ cell lines depleted for endogenous CTCF. The ATAC-seq signal intensity at the CTCF binding peaks or genome-wide scale in CTCF^AID2/dRBR^ cells was similar to CTCF^AID2/WT^ (Fig. 4A, Additional file 1: Fig. S4 A, Additional file 3: Table S2). The Spearman’s correlation also showed that the ATAC-seq signal pattern clustered closely between CTCF-dRBR and CTCF-WT (Fig. 4B, Additional file 1: Fig. S4B). No ATAC-seq signal changes were observed at the locus of CTCF target genes *MYC* and *RBM45* (Fig. 4C–D). The differential ATAC-seq peaks were called between CTCF-dRBR vs CTCF-WT based on different cutoffs. At the most stringent cutoff (FDR < 0.05 and FC > 2), ten downregulated peaks and three upregulated peaks were found among > 100k ATAC-seq peaks genome-wide. When a lower cutoff was applied, a significant number of differential peaks were observed particularly by switching FDR to *P* value. However, the concern of reliability arises as well (Additional file 1: Fig. S4 C). In summary, our data suggests that RNA interaction with CTCF plays a limited role in regulating genome-wide chromatin accessibility.

Previously, we and others observed that global gene expression was mildly affected by acute depletion of total endogenous CTCF [1, 3, 11]. Here, we investigated the transcriptional change between cells expressing CTCF-dRBR or CTCF-WT without endogenous CTCF. Total RNA-seq was conducted twice, once with two replicates of RNA collected from CTCF^AID2/WT^ and CTCF^AID2/dRBR^ cells and again with three replicates. The first assay of two replicates showed only three genes demonstrated expression changes at the cutoff of FDR < 0.05 (Additional file 1: Fig. S4D–E, Additional file 4: Table S3). To improve statistical significance, the RNA-seq was repeated with three replicates. Using three replicates and a cutoff of FDR < 0.05, no genes were found to be differentially expressed between groups. Two well-characterized CTCF targets in SEM cells, *RBM45* and *MYC*, had no significant transcription changes (Fig. 4E–F, Additional file 1: Fig. S4 F). Collectively, these data suggest that loss of the RNA interaction region of CTCF (RBR) plays a limited role in its DNA binding for gene regulation.

## Discussion

Protein and RNA interactions have been proposed to be critical in many biological processes, such as splicing regulation, mRNA transport, RNA stability maintenance, RNA trafficking, and mRNA translation. However, it is worth noting that although locus-specific RNAs, such as lncRNAs or eRNAs, were reported to play a critical role in transcription in a *cis*-acting manner, most of the RNA-interaction results in CTCF regulation are *in trans* RNAs. Given RNAs’ lack of consensus motifs for CTCF protein to search and precisely bind, the biological consequence of these *in trans* distributed RNAs requires cautious interpretation. Additionally, protocols to identify RNA–protein interactions produce inconsistent results, and data analysis among the techniques is variable. For example, CLAP-seq [19] did not recapitulate most RNAs identified by CLIP-seq to bind CTCF. Therefore, it is challenging to directly compare these protocols developed by different people under various conditions.

Previous studies by Saldana-Meyer et al. and Hansen et al. showed RNAs were essential to CTCF-mediated genome organization [13, 18]. Saldana-Meyer et al. utilized the auxin-inducible degradation system to degrade endogenous CTCF in mESCs while exogenously expressing WT CTCF and mutants of the putative RNA binding regions, ∆ZF1 and ∆ZF10. PAR-CLIP showed reduced RNA binding to CTCF in cells expressing ∆ZF1 and ∆ZF10. Differential chromatin binding, gene expression, and chromatin looping were observed in both mutants when compared to cells expressing WT CTCF. In addition, RNA was disrupted by triptolide and RNase A in mESCs and showed modest changes to global CTCF chromatin occupancy. However, for all ChIP-seq experiments spike-in normalization was variable among samples. Of note, the auxin degradation system available at the time of the study was the original AID1 design, which requires high amounts of auxin to achieve protein degradation. High concentrations of auxin have since been shown to cause significant cellular toxicity that could cloud data interpretation [11, 23]. Hansen et al. replaced the RBR of CTCF in mESCs with a linker and 3xHA tag to generate an endogenous RBR mutant, ∆RBR_i_-CTCF. ChIP-seq using spike-in normalization revealed a global decrease in CTCF chromatin occupancy and disrupted chromatin looping of some sites that were designated to be dependent on the RBR. The authors noted protein stability of the ∆RBR_i_-CTCF protein was compromised compared to WT and questioned whether the reduced expression resulted in fewer chromatin contacts. Additionally, ∆RBR_i_-CTCF cells doubling time was greater when compared to WT-CTCF cells. In contrast, we observed equal CTCF protein expression between CTCF^AID2/WT^ and CTCF^AID2/dRBR^ cell lines, and there were no changes to cellular fitness. While these studies provide important insights into the significance of CTCF-RNA interactions, the studies were limited to mESCs and used varied methodologies and analyses, making direct comparisons to our study challenging (Additional file 5: Table S4).

To characterize the role of RNAs on CTCF-DNA binding, chromatin accessibility, and transcription, our study focused on assaying the downstream effects caused by perturbation of CTCF-RNA interactions to determine the biological impact of the CTCF-RNA interaction axis. Our study demonstrated three independent and complementary approaches to evaluate the potential implications of CTCF-RNA interactions on DNA binding, transcription regulation, and chromatin accessibility. While HA-tagged CTCF-ChIP-seq demonstrated limited changes in the dRBR setting, the reduced peak number and density from HA-tagged ChIP-seq experiments might bias on target selection. To this end, we optimized our treatment protocol and conducted CTCF antibody-based ChIP-seq. Indeed, we observed a fraction of differential CTCF binding peaks with the authentic consensus CTCF binding motif in RNase A and triptolide treatment samples, respectively. Interestingly, in both settings, much more upregulated CTCF-binding peaks were identified upon RNA depletion with a lower statistic cutoff (*P* value instead of FDR). Given that it is impossible to evaluate the transcriptional impact of CTCF-RNA interactions in triptolide and RNase A treatment groups, our transcriptome analysis heavily relied on the dRBR mutant form reported by others. Although in vitro biochemistry characterization by Hansen et al. clearly showed the reduction of RNA interaction in dRBR, it is still unknown whether other RBR-binding domains are required in our model system.

## Conclusions

This study concludes that CTCF-RNA interactions do not shape global CTCF-DNA interactions but confer variable effects at selective loci. Locus-dependent targeting approaches would be beneficial to investigate the function of CTCF-RNA interactions in the future. Additionally, the RNA-binding affinity of CTCF may play a role in a tissue-specific manner that could be investigated by more model systems. Moreover, this field urgently requires more technological innovations to promote the investigation of RNA–protein interactions further.

## Methods

### Cell culture

The SEM B-ALL cell line from DSMZ was used to make the CTCF^AID2^, CTCF^AID2/WT^, and CTCF^AID2/dRBR^ cell lines. These were previously described [11]. In brief, CTCF^AID2^ cells have an in-frame miniAID-mClover3 tag at the C-terminus of endogenous CTCF and constitutively express OsTIR1 F74G (Addgene 232800). The CTCF^AID2/WT^ and CTCF^AID2/dRBR^ cells were CTCF^AID2^ cells transduced with the doxycycline-inducible wild-type HA-tagged CTCF (Addgene 232801) and the HA-tagged CTCF-dRBR mutant (Addgene 232802), respectively. All SEM cells and derivative lines were cultured in RPMI- 1640 medium (Lonza) containing 10% fetal bovine serum (Hyclone), 2 mM glutamine (Sigma), and 1% penicillin/streptomycin (Thermo Fisher Scientific) in an incubator at 37 °C, 5% CO_2_, and 95% humidity. To swap from endogenous CTCF-miniAID-mClover to exogenous HA-tagged CTCF expression, one or 10 µM 5-Ph-IAA (MedChemExpress) was used to induce the degradation of CTCF-miniAID-mClover in CTCF^AID2/WT^ and CTCF^AID2/dRBR^ cells by a 6-h treatment before induction of exogenous HA-tagged CTCF with 1 µg/mL doxycycline for 18 h concurrent with continuous 5-Ph-IAA treatment. All cell lines were validated by STR and verified to be free of mycoplasma by Lookout Mycoplasma PCR Detection Kit (Sigma, #MP0035).

### Immunoblotting

Cells were lysed in RIPA buffer, and lysates were run on an SDS-PAGE gel (Thermo Fisher Scientific). Protein was transferred to a PVDF membrane (Bio-Rad) at 100 V for 1 h. Membranes were blocked with 5% non-fat milk in TBS-T (10 mM Tris, pH 8.0, 150 mM NaCl, 0.5% Tween- 20) for 1 h at room temperature before incubating with target antibodies overnight at 4 °C with gentle rocking (GAPDH, Thermo Fisher Scientific, AM4300, 1:10,000; CTCF, Santa Cruz, sc- 271514, 1:200; CTCF, Diagenode, C15410210 - 50, 1:2000). Following three 10-min washes in TBS-T, membranes were incubated with a 1:2000 (CTCF, Santa Cruz) or 1:20,000 (GAPDH) dilution of sheep anti-mouse IgG HRP (GE Healthcare, NA931) or 1:5000 (CTCF, Diagenode) dilution of donkey anti-rabbit IgG HRP (GE Healthcare, NA934) in 5% non-fat milk/TBS-T for 1 h at room temperature. Blots were developed with ECL (Perkin Elmer) and visualized by Licor System.

### ChIP-seq

For all ChIP experiments, the switch from endogenous CTCF-miniAID-mClover to exogenous HA-tagged CTCF was described in the cell culture methods. For HA-CTCF ChIP with triptolide treatment, 20 million CTCF^AID2/WT^ cells were treated with either DMSO or 10 μM triptolide (Cayman Chemicals) for 4 h in triplicate concurrent with 5-Ph-IAA and doxycycline (Fig. 2A). Cells were fixed with 1% formaldehyde for 5 min with gentle rocking at room temperature, and chromatin was prepared using the Covaris TruChIP Chromatin Shearing Kit (Covaris, 520154). Chromatin was sheared by the Covaris M220 ultrasonicator (duty factor of 10, cycles/burst 200 for 10 min at set point 6 °C). After clarification by centrifugation at 8000 × g for 10 min, the chromatin was moved to a new 1.5-mL Eppendorf tube, and the buffer was amended for immunoprecipitation (final concentration: 50 mM Tris HCL pH 7.4, 100 mM NaCl, 1 mM EDTA, 1% SDS, 0.5% Na deoxycholate plus protease inhibitors). Chromatin was incubated with anti-HA magnetic beads (Pierce) at 4 °C overnight with gentle rotation. On a magnetic stand, the beads were washed twice with wash buffer 1 (50 mM Tris HCL pH 7.4, 1 M NaCl, 1 mM EDTA, 1% NP- 40, 0.1% SDS, 0.5% Na deoxycholate plus protease inhibitors) and once with wash buffer 2 (20 mM Tris HCL pH 7.4, 10 mM MgCl_2_, 0.2% Tween- 20 plus protease inhibitors). The beads in wash buffer 2 were transferred to a new 1.5-mL Eppendorf tube and placed on a magnetic stand to remove the wash buffer. Decrosslinking was carried out in 1X TE plus 1% SDS, proteinase K, and 400 mM NaCl at 65 °C for 4 h, followed by phenol, chloroform, and isopropyl alcohol precipitation of the DNA. NEBNext Ultra II NEB Library Prep Kit and NEBNext Multiplex oligos for Illumina were used to construct libraries for sequencing. For HA-CTCF ChIP with RNase A treatment, 20 million CTCF^AID2/WT^ cells were grown in triplicate. RNase A (5 μg/mL) was added to the chromatin along with the anti-HA magnetic beads for overnight incubation at 4 °C with gentle rotation (Fig. 2A). For CTCF-ChIP (Fig. 3A), CTCF^AID2/WT^ cells were treated with 1 µM triptolide or DMSO for 8 h concurrent with 5-Ph-IAA and doxycycline treatment. For RNase A ChIP, RNase A treatment conditions were adapted from those previously reported [13, 25]. Cells were permeabilized before fixation by 0.05% TWEEN- 20 in PBS for 10 min on ice, washed once with PBS, and resuspended in PBS plus 1 mg/mL RNase A or mock-treated and rotated for 45 min at room temperature. Each treatment group (+ RNase A, − RNase A) was performed in triplicate. For both triptolide and RNase A ChIP groups, chromatin was prepared as described above. Spike-in antibody and chromatin (Active Motif, 61686 and 53083) along with CTCF antibody (10 µg, Diagenode, C15410210 - 50) were added to the chromatin and incubated at 4 °C overnight with gentle rotation. The next day, pre-washed Protein G beads (Dynabeads, Invitrogen, 10004D) were added and incubated at 4 °C for 4 h with gentle rotation. Washes and elution of DNA were as described above.

### RNA-seq

Trizol (Thermo Fisher Scientific, 15596026) extraction was used to isolate RNA from CTCF^AID2/WT^ and CTCF^AID2/dRBR^ cells treated with 5-Ph-IAA for 6 h to degrade endogenous CTCF-miniAID-mClover and 18 h doxycycline to induce HA tagged CTCF concurrent with 5-Ph-IAA. The Kapa RNA HyperPrep Kit with RiboErase (HMR) was used to prepare cDNA libraries.

### Quantitative real-time PCR

The High-Capacity cDNA Reverse Transcriptase kit (Applied Biosystems, 4374966) was used to make cDNA, and FAST SYBR Green Master Mix was used for real-time qPCR (Applied Biosystems, 4385612) with primers to amplify *CTCF*, *MYC*, *RBM45*, and *GAPDH*. CTCF-F: 5′ TTTGTCTGTTCTAAGTGTGGGAAA3′, CTCF-R: 5′TTAGAGCGCATCTTTCTTTTTCTT- 3′; MYC-F: 5′TCAAGAGGTGCCACGTCTCC3′, MYC-R: 5′TCTTGGCAGCAGGATAGTCCTT3′; RBM45-F: 5′TCACCGAGATGTTGAAGATGA3′, RBM45-R: 5′TCGTACGTAGCCCAAACCTT3′; GAPDH-F: 5′AGGGCTGCTTTTAACTCTGGT3′, GAPDH-R: 5′CCCCACTTGATTTTGGAGGGA 3′. The ^ΔΔ^CT method was used to determine relative expression levels [26].

### ATAC-seq

ATAC-seq was performed following the protocol described previously [27]. Nuclei were isolated from 75,000 cells in duplicate for each sample. Cell pellets were resuspended in 150 µL cold ATAC-RSB +++ buffer (10 mM Tris pH 7.4, 10 mM NaCl, 3 mM MgCl_2_, 0.1% NP- 40, 0.1% TWEEN20, 0.01% digitonin and protease inhibitors) and incubated on ice for 3 min. One milliliter ATAC-RSB + buffer (10 mM Tris pH 7.4, 10 mM NaCl, 3 mM MgCl_2_, 0.1% TWEEN20, and protease inhibitors) was added, and samples were centrifuged at 500 rpm for 10 min at 4 °C. The nuclear pellets were resuspended in a 50 µL reaction buffer [final concentration 1X Tagment DNA Buffer (Nextera, FC- 121–1030), 2.5 µL Nextera Tn5 (Nextera, FC- 121–1030)] and incubated at 37 °C for 30 min with 1000 rpm shaking. The Qiagen MinElute PCR purification kit (Qiagen, 28004) was used to purify DNA, and indexing PCR was carried out for 12 cycles with NEBNext HiFi 2X PCR Master Mix (NEB, M0541S) and indexing primers [28]. Agencourt AMPure XP beads (Beckman Coulter, A63881) were used at a 1:3 ratio to purify DNA.

### RNA-seq data analysis

We performed the paired-end 101-cycle sequencing on the NovaSeq 6000 sequencer (Illumina) and analyzed the data using a standard pipeline. Briefly, raw reads were trimmed using TrimGalore (v0.6.3, “–paired –retain_unpaired”) and aligned to the *Homo sapiens* reference genome GRCh37.p13(hg19) using STAR (v2.7.9a) [29]. Gene-level read quantification was performed using RSEM (v1.3.1) on the Gencode annotation v19 [30]. Differential gene expression analysis was conducted using the TMM normalization method (genes with CPM < 1 in all samples were removed) followed by Limma-voom analysis using the “voom,” “lmFit,” and “eBayes” functions from the limma R package [31].

### ChIP-seq data analysis

We performed single-end 51-cycle sequencing on the NovaSeq 6000 sequencer (Illumina). For HA-tag-ChIP-Seq samples, raw reads were trimmed using TrimGalore (v0.6.3) and aligned to the *Homo sapiens* reference genome GRCh37.p13(hg19) using BWA (v0.7.17-r1198). Duplicated and low mapping quality reads were removed using the “bamsormadup” function from the biobambam2 tool (v2.0.87) and samtools (version 1.9, parameter “-q 1 -F 1024”) [32]. The fragment size in each sample was estimated based on the cross-correlation profile calculated from SPP (v1.11). Fragments were extended to fragment size and normalized to 15 million reads to generate bigwig files. Macs2 was used to call peaks using parameters “-g hs –nomodel –extsize < SPP_fragmentSize >.” For spike-in CTCF ChIP-Seq samples, the reads were first aligned to a hybrid-genome constructed from the human GRCh37.p13 genome and the *Drosophila melanogaster* (dm6) after trimming. Human and *Drosophila* reads were then extracted into two separated bam files, and the subsequent analyses followed the same workflow as described above. To identify differential peaks, the reference peaks used for comparisons were generated as follows: For each sample, both “high confidence peaks” (parameter “-q 0.05”) and “low confidence peaks” (parameter “-q 0.5”) were called. Reproducible peaks (called as a high confidence peak in at least one replicate that also overlapped with a low confidence peak in the other replicates) were generated for each cell type. For HA-tag-ChIP-Seq samples, the union of reproducible peaks across cell types was used as reference peaks. For spike-in CTCF ChIP-Seq samples, reproducible peaks within each comparison group were combined as reference peaks for paired comparison. For each reference peak, the number of overlapping ChIP-seq fragments was counted. In spike-in samples, read counts within reference peaks were normalized using spike-in scaling factors as follows: The spike-in read counts for each sample were first normalized by dividing the maximum spike-in read counts across all samples. Then, scaling factors were computed by dividing these normalized values by the geometric mean of all normalized values. The read counts in reference peaks were adjusted by the calculated scaling factors. Differential peaks were identified using the empirical Bayes method from the limma R package. For downstream analyses, both heatmaps and Spearman’s correlation were generated by deepTools [33].

### ATAC-seq data analysis

We performed paired-end 101-cycle sequencing on the NovaSeq 6000 sequencer (Illumina). The reads were trimmed for the Nextera adapter by cutadapt (v1.9, paired-end mode, default parameter with “-m 6 -O 20”) and aligned to the human genome hg19(GRCh37-lite) by BWA (v0.7.12-r1039, default parameter) [34]. The duplicated reads were then marked with biobambam2 (v2.0.87), and only nonduplicated proper paired reads were kept by samtools (parameter “-q 1 -F 1804,” v1.2) [32]. After removing the mitochondrial DNA reads, the rest were classified into four groups, including nucleosome-free reads and nucleosome reads by fragment size. The bigwig files were generated using the center 80-bp fragments and scaled to 20 million nucleosome-free reads. We observed reasonable nucleosome-free peaks and patterns of nucleosome peaks surrounding the nucleosome-free peaks on the Integrative Genomics Viewer (Broad Institute). All samples exhibited double the ENCODE criteria. Therefore, we concluded the data showed enough depth. Given that all samples exhibited more than 20 million fragments, we were confident that most strong peaks were not missed. Peak calling on the nucleosome-free reads was conducted by MACS2 (v2.1.1.20160309, default parameters with “–extsize 200 -nomodel”) [35]. To assure replicability, we first finalized the reproducible peaks for each group as only a retained peak if it was called with a stringent cutoff (macs2 -q 0.05) in one merged sample and was at least called with a lower cutoff (macs2 -q 0.5) in the other merged sample. The reproducible peaks were further merged between the groups to create a final set of reference chromatin-accessible regions. We then counted the nucleosome-free reads from each sample overlapping the reference regions by bedtools (v2.24.0). The reproducibility was optimal because Spearman’s correlation coefficient between the replicates was > 0.9 and more significant than the between-sample variability from different groups. To elucidate the differentially accessible regions (DARs), we normalized the raw nucleosome-free read counts used to trim the mean of the M-value normalization method. We applied empirical Bayes statistical tests after linear fitting from the voom package (R 3.23, edgeR 3.12.1, limma 3.26.9) [31]. When there are no DARs defined by FDR-corrected *P* value < 0.05 (Benjamini–Hochberg procedure) and fold change > 2, we relax the cutoff to *P* value < 0.05 and fold change > 2 for the UpSet plot and heatmap.

## Supplementary Information

Additional file 1. Supplementary Figures S1–S4.

Additional file 2. Table S1 Statistical overview of ChIP-seq data analysis.

Additional file 3. Table S2 Reproducible ATAC-seq peak information

Additional file 4. Table S3 Data summary of RNA-seq analysis collected in this study.

Additional file 5. Table S4 Statistical analysis across different studies.

Additional file 6. Q-PCR raw data.

Additional file 7. Immunoblotting raw data.

Additional file 8. Review history.

## Data Availability

Data generated in this study, including total RNA-seq, ChIP-seq, and ATAC-seq, were deposited at NCBI GEO as GSE261176, GSE261179, GSE281633, and GSE288854 [36–39] for public access. Additional files 2–4: Tables S1–S3 summarize processed data for ChIP-seq peak, ATAC-seq peak, and RNA-expression profiling. Code repositories collected at figshare included RNA-seq, ChIP-seq, and ATAC-seq [40]. Previously public data used in this study includes GSE205311 and GSE205408 [41, 42].

## References

[1] Hyle J, Zhang Y, Wright S, Xu B, Shao Y, Easton J, Tian L, Feng R, Xu P, Li C. Acute depletion of CTCF directly affects MYC regulation through loss of enhancer-promoter looping. Nucleic Acids Res. 2019;47:6699–713. 31127282 10.1093/nar/gkz462 PMC6648894

[2] Xu B, Wang H, Wright S, Hyle J, Zhang Y, Shao Y, Niu M, Fan Y, Rosikiewicz W, Djekidel MN, et al. Acute depletion of CTCF rewires genome-wide chromatin accessibility. Genome Biol. 2021;22:244. 34429148 10.1186/s13059-021-02466-0 PMC8386078

[3] Nora EP, Goloborodko A, Valton AL, Gibcus JH, Uebersohn A, Abdennur N, Dekker J, Mirny LA, Bruneau BG. Targeted degradation of CTCF decouples local insulation of chromosome domains from genomic compartmentalization. Cell. 2017;169:930-944.e922. 28525758 10.1016/j.cell.2017.05.004 PMC5538188

[4] Lobanenkov VV, Nicolas RH, Adler VV, Paterson H, Klenova EM, Polotskaja AV, Goodwin GH. A novel sequence-specific DNA binding protein which interacts with three regularly spaced direct repeats of the CCCTC-motif in the 5′-flanking sequence of the chicken c-myc gene. Oncogene. 1990;5:1743–53. 2284094

[5] Klenova EM, Nicolas RH, Paterson HF, Carne AF, Heath CM, Goodwin GH, Neiman PE, Lobanenkov VV. CTCF, a conserved nuclear factor required for optimal transcriptional activity of the chicken c-myc gene, is an 11-Zn-finger protein differentially expressed in multiple forms. Mol Cell Biol. 1993;13:7612–24. 8246978 10.1128/mcb.13.12.7612-7624.1993 PMC364833

[6] Ong C-T, Corces VG. CTCF: an architectural protein bridging genome topology and function. Nat Rev Genet. 2014;15:234–46. 24614316 10.1038/nrg3663 PMC4610363

[7] Ong C-T, Corces VG. Enhancer function: new insights into the regulation of tissue-specific gene expression. Nat Rev Genet. 2011;12:283–93. 21358745 10.1038/nrg2957 PMC3175006

[8] Phillips JE, Corces VG. CTCF: master weaver of the genome. Cell. 2009;137:1194–211. 19563753 10.1016/j.cell.2009.06.001 PMC3040116

[9] Pombo A, Dillon N. Three-dimensional genome architecture: players and mechanisms. Nat Rev Mol Cell Biol. 2015;16:245–57. 25757416 10.1038/nrm3965

[10] Rowley MJ, Corces VG. Organizational principles of 3D genome architecture. Nat Rev Genet. 2018;19:789–800. 30367165 10.1038/s41576-018-0060-8 PMC6312108

[11] Hyle J, Djekidel MN, Williams J, Wright S, Shao Y, Xu B, Li C. Auxin-inducible degron 2 system deciphers functions of CTCF domains in transcriptional regulation. Genome Biol. 2023;24:14. 36698211 10.1186/s13059-022-02843-3 PMC9878928

[12] Oksuz O, Henninger JE, Warneford-Thomson R, Zheng MM, Erb H, Vancura A, Overholt KJ, Hawken SW, Banani SF, Lauman R, et al. Transcription factors interact with RNA to regulate genes. Mol Cell. 2023;83(2449–2463):e2413. 10.1016/j.molcel.2023.06.012 PMC10529847 37402367

[13] Saldana-Meyer R, Rodriguez-Hernaez J, Escobar T, Nishana M, Jacome-Lopez K, Nora EP, Bruneau BG, Tsirigos A, Furlan-Magaril M, Skok J, Reinberg D. RNA interactions are essential for CTCF-mediated genome organization. Mol Cell. 2019;76(412–422):e415. 10.1016/j.molcel.2019.08.015 PMC7195841 31522988

[14] Oh HJ, Aguilar R, Kesner B, Lee HG, Kriz AJ, Chu HP, Lee JT. Jpx RNA regulates CTCF anchor site selection and formation of chromosome loops. Cell. 2021;184(6157–6173):e6124. 10.1016/j.cell.2021.11.012 PMC8671370 34856126

[15] Kung JT, Kesner B, An JY, Ahn JY, Cifuentes-Rojas C, Colognori D, Jeon Y, Szanto A, del Rosario BC, Pinter SF, et al. Locus-specific targeting to the X chromosome revealed by the RNA interactome of CTCF. Mol Cell. 2015;57:361–75. 25578877 10.1016/j.molcel.2014.12.006 PMC4316200

[16] Wei C, Jia L, Huang X, Tan J, Wang M, Niu J, Hou Y, Sun J, Zeng P, Wang J, et al. CTCF organizes inter-A compartment interactions through RYBP-dependent phase separation. Cell Res. 2022;32:744–60. 35768498 10.1038/s41422-022-00676-0 PMC9343660

[17] Lee R, Kang MK, Kim YJ, Yang B, Shim H, Kim S, Kim K, Yang CM, Min BG, Jung WJ, et al. CTCF-mediated chromatin looping provides a topological framework for the formation of phase-separated transcriptional condensates. Nucleic Acids Res. 2022;50:207–26. 34931241 10.1093/nar/gkab1242 PMC8855298

[18] Hansen AS, Hsieh TS, Cattoglio C, Pustova I, Saldana-Meyer R, Reinberg D, Darzacq X, Tjian R. Distinct classes of chromatin loops revealed by deletion of an RNA-binding region in CTCF. Mol Cell. 2019;76(395–411):e313. 10.1016/j.molcel.2019.07.039 PMC7251926 31522987

[19] Guo JK, Blanco MR, Walkup WGt, Bonesteele G, Urbinati CR, Banerjee AK, Chow A, Ettlin O, Strehle M, Peyda P, et al. Denaturing purifications demonstrate that PRC2 and other widely reported chromatin proteins do not appear to bind directly to RNA in vivo. Mol Cell. 2024;84(1271-1289):e12 10.1016/j.molcel.2024.01.026 PMC10997485 38387462

[20] Lee Y, Blum R, Rosenberg M, Lee JT. Re-analysis of CLAP data affirms PRC2 as an RNA binding protein. bioRxiv. 2024.

[21] Titov DV, Gilman B, He QL, Bhat S, Low WK, Dang Y, Smeaton M, Demain AL, Miller PS, Kugel JF, et al. XPB, a subunit of TFIIH, is a target of the natural product triptolide. Nat Chem Biol. 2011;7:182–8. 21278739 10.1038/nchembio.522 PMC3622543

[22] Wang Y, Lu JJ, He L, Yu Q. Triptolide (TPL) inhibits global transcription by inducing proteasome-dependent degradation of RNA polymerase II (Pol II). PLoS ONE. 2011;6: e23993. 21931633 10.1371/journal.pone.0023993 PMC3172214

[23] Shao W, Zeitlinger J. Paused RNA polymerase II inhibits new transcriptional initiation. Nat Genet. 2017;49:1045–51. 28504701 10.1038/ng.3867

[24] Yesbolatova A, Saito Y, Kitamoto N, Makino-Itou H, Ajima R, Nakano R, Nakaoka H, Fukui K, Gamo K, Tominari Y, et al. The auxin-inducible degron 2 technology provides sharp degradation control in yeast, mammalian cells, and mice. Nat Commun. 2020;11:5701. 33177522 10.1038/s41467-020-19532-z PMC7659001

[25] Beltran M, Yates CM, Skalska L, Dawson M, Reis FP, Viiri K, Fisher CL, Sibley CR, Foster BM, Bartke T, et al. The interaction of PRC2 with RNA or chromatin is mutually antagonistic. Genome Res. 2016;26:896–907. 27197219 10.1101/gr.197632.115 PMC4937559

[26] Schmittgen TD, Livak KJ. Analyzing real-time PCR data by the comparative C(T) method. Nat Protoc. 2008;3:1101–8. 18546601 10.1038/nprot.2008.73

[27] Corces MR, Trevino AE, Hamilton EG, Greenside PG, Sinnott-Armstrong NA, Vesuna S, Satpathy AT, Rubin AJ, Montine KS, Wu B, et al. An improved ATAC-seq protocol reduces background and enables interrogation of frozen tissues. Nat Methods. 2017;14:959–62. 28846090 10.1038/nmeth.4396 PMC5623106

[28] Buenrostro JD, Giresi PG, Zaba LC, Chang HY, Greenleaf WJ. Transposition of native chromatin for fast and sensitive epigenomic profiling of open chromatin, DNA-binding proteins and nucleosome position. Nat Methods. 2013;10:1213–8. 24097267 10.1038/nmeth.2688 PMC3959825

[29] Dobin A, Davis CA, Schlesinger F, Drenkow J, Zaleski C, Jha S, Batut P, Chaisson M, Gingeras TR. STAR: ultrafast universal RNA-seq aligner. Bioinformatics. 2013;29:15–21. 23104886 10.1093/bioinformatics/bts635 PMC3530905

[30] Harrow J, Frankish A, Gonzalez JM, Tapanari E, Diekhans M, Kokocinski F, Aken BL, Barrell D, Zadissa A, Searle S, et al. GENCODE: the reference human genome annotation for the ENCODE Project. Genome Res. 2012;22:1760–74. 22955987 10.1101/gr.135350.111 PMC3431492

[31] Law CW, Chen Y, Shi W, Smyth GK. voom: precision weights unlock linear model analysis tools for RNA-seq read counts. Genome Biol. 2014;15:R29. 24485249 10.1186/gb-2014-15-2-r29 PMC4053721

[32] Li H, Handsaker B, Wysoker A, Fennell T, Ruan J, Homer N, Marth G, Abecasis G, Durbin R. Genome Project Data Processing S: The Sequence Alignment/Map format and SAMtools. Bioinformatics (Oxford, England). 2009;25:2078–9. 19505943 10.1093/bioinformatics/btp352 PMC2723002

[33] Ramirez F, Ryan DP, Gruning B, Bhardwaj V, Kilpert F, Richter AS, Heyne S, Dundar F, Manke T. deepTools2: a next generation web server for deep-sequencing data analysis. Nucleic Acids Res. 2016;44:W160-165. 27079975 10.1093/nar/gkw257 PMC4987876

[34] Li H, Durbin R. Fast and accurate short read alignment with Burrows-Wheeler transform. Bioinformatics (Oxford, England). 2009;25:1754–60. 19451168 10.1093/bioinformatics/btp324 PMC2705234

[35] Zhang Y, Liu T, Meyer CA, Eeckhoute J, Johnson DS, Bernstein BE, Nusbaum C, Myers RM, Brown M, Li W, Liu XS. Model-based analysis of ChIP-Seq (MACS). Genome Biol. 2008;9:R137. 18798982 10.1186/gb-2008-9-9-r137 PMC2592715

[36] Hyle J, Nadhir Djekidel M, Rosikiewicz W, Xu B, Li C. Deciphering the role of RNA in regulating CTCF’s DNA binding affinity in leukemia cells [CTCF-HA-ChIP]. 2024. https://www.ncbi.nlm.nih.gov/geo/query/acc.cgi?acc=GSE261176. GSE261176 10.1186/s13059-025-03582-x PMC12067947 40355969

[37] Hyle J, Nadhir Djekidel M, Rosikiewicz W, Xu B, Li C. Deciphering the role of RNA in regulating CTCF’s DNA binding affinity in leukemia cells [ATAC-seq]. 2024. https://www.ncbi.nlm.nih.gov/geo/query/acc.cgi?acc=GSE261179. GSE261179. 10.1186/s13059-025-03582-x PMC12067947 40355969

[38] Hyle J, Nadhir Djekidel M, Rosikiewicz W, Xu B, Li C. Deciphering the role of RNA in regulating CTCF’s DNA binding affinity in leukemia cells [CTCF-ChIP]. 2024. https://www.ncbi.nlm.nih.gov/geo/query/acc.cgi?acc=GSE281633. GSE281633. 10.1186/s13059-025-03582-x PMC12067947 40355969

[39] Hyle J, Nadhir Djekidel M, Rosikiewicz W, Xu B, Li C. Deciphering the role of RNA in regulating CTCF’s DNA binding affinity in leukemia cells [RNA-Seq.New]. 2024. https://www.ncbi.nlm.nih.gov/geo/query/acc.cgi?acc=GSE288854. GSE288854. 10.1186/s13059-025-03582-x PMC12067947 40355969

[40] Xu B, Djekidel N, Li C, Williams J. St Jude Center for Applied Bioinformatics General Pipelines. figshare. Collection. 2022. 10.6084/m9.figshare.c.6186670 .

[41] Hyle J, Nadhir Djekidel M, Williams J, Wright S, Shao Y, Xu B, Li C. Auxin-inducible degron 2 system deciphers functions of CTCF domains in transcriptional regulation [RNA-Seq]. 2022. https://www.ncbi.nlm.nih.gov/geo/query/acc.cgi?acc=GSE205311 . GSE205311. 10.1186/s13059-022-02843-3 PMC9878928 36698211

[42] Hyle J, Nadhir Djekidel M, Williams J, Wright S, Shao Y, Xu B, Li C. Advanced auxin-inducible degron system deciphers unique functions of CTCF in transcriptional regulation [ChIP-seq]. 2022. https://www.ncbi.nlm.nih.gov/geo/query/acc.cgi?acc=GSE205408 . GSE205408. 10.1186/s13059-022-02843-3 PMC9878928 36698211

