# Supplementary material for: Deciphering the role of RNA in regulating CTCF’s DNA binding affinity in leukemia cells
Source: Genome Biol. 2025 May 12;26:126. doi: 10.1186/s13059-025-03582-x (PMC12067947; doi:10.1186/s13059-025-03582-x)
Supplement: Supplementary file 1 — Additional file 1. Supplementary Figures S1–S4. [file 13059_2025_3582_MOESM1_ESM.pdf]

Additional File 1 Figure Legends

Additional File 1: Fig. S1. Quality control of HA-CTCF ChIP-seq in the presence of RNA-binding deficiency or RNA depletion.

(**A**) Schematic diagram illustrating how the ectopic HA-tagged CTCF swap system works in combination with acute protein degradation of endogenous CTCF. The homozygous miniAID-mClover3 knockin SEM cell line, $CTCF^{AID2}$, was generated in our previous study [11]. The 5-Ph-IAA auxin analog is added to cell culture for 6 hours, binds to the miniAID tag (fused to endogenous CTCF protein) and recruits binding to the OsTIR1(F74G) protein and the SCF complex, which ubiquitinates the CTCF protein leading to acute degradation. Concurrently, the CTCF HA-tagged WT or RNA-binding region deficient form is induced by doxycycline for an additional 18 hours.

(**B**) Immunoblot analysis of endogenous ($CTCF^{AID2.0}$) and induced exogenous (HA-CTCF) expression of CTCF using an antibody for CTCF. $CTCF^{AID2.0}$ expression can be seen in all untreated samples (-, -). After 6 hours of 10 μM 5-Ph-IAA treatment, $CTCF^{AID2}$ protein expression is degraded. Exogenous expression of HA-tagged CTCF wildtype and dRBR mutant is comparable to endogenous CTCF following 18 hours of 1 μg/mL doxycycline with concurrent 10 μM 5-Ph-IAA treatment (+, +). GAPDH was included as a loading control. The dZF1 and dZF10 mutant forms of CTCF were included as positive blotting controls to indicate the accurate molecular weight.

(**C**) Reproducible HA ChIP-seq signals were used from $CTCF^{AID2/WT}$ cells and $CTCF^{AID2/dRBR}$ cells, Triptolide, RNase A, and DMSO treatment groups to conduct the Spearman's correlation, which was calculated to quantify the overall similarity between samples.

(**D**) Violin plot of the peak score of ChIP-seq from CTCF-antibody only peaks and shared peaks between HA and CTCF-antibody pulldown.

Additional File 1: Fig. S2. Quality control of Triptolide and RNase A treatment conditions for CTCF ChIP-seq.

(**A**) Q-PCR was conducted to quantify the transcription decrease of *MYC*, *RBM45*, and *CTCF* in response to Triptolide treatment (4 hours) in a dosage-dependent manner.

(**B**) Q-PCR was conducted to quantify the transcription decrease of *MYC*, *RBM45*, and *CTCF* in response to Triptolide treatment (0.1 and 1 μM) in a time-course manner.

(**C**) Dead cell % quantification was conducted in cells in response to Triptolide treatment (0.1 and 1 μM) in a time-course manner.

(**D**) Q-PCR was conducted to quantify the transcription decrease of *MYC* (short half-life) and *GAPDH* (long half-life) in response to RNase A treatment (1mg/mL) in a time-course manner.

(**E**) In the pre-fixation RNase A treatment group, a pilot fraction of samples was collected for Q-PCR against *MYC*, *RBM45*, *CTCF,* and *GAPDH* to confirm the RNA depletion efficiency.

(**F**) Replicate ChIP-seq samples were tested for immunoprecipitation efficiency by CTCF-antibody mediated pull-down and immunoblotting against CTCF in each RNase A treatment and control group. I: Input; FT: flow-through fraction; E: elution fraction.

(**G**) Replicate ChIP-seq samples were tested for immunoprecipitation efficiency by CTCF-antibody mediated pull-down and immunoblotting against CTCF in each Triptolide treatment and DMSO group. I: Input; FT: flow-through fraction; E: elution fraction.

Additional File 1: Fig. S3. CTCF ChIP-seq analysis for CTCF-RNA interaction interference.

(**A**) Reproducible CTCF ChIP-seq signals were used from Triptolide and DMSO treatment groups to conduct Spearman's correlation, which was calculated to quantify the overall similarity between samples.

(**B**) Reproducible CTCF ChIP-seq signals were used from + RNase A and no treatment groups to conduct Spearman's correlation, which was calculated to quantify the overall similarity between samples.

(**C**) Genomic heatmap of total reproducible genome-wide CTCF-binding peak signals centered at peak midpoint from Triptolide and DMSO treatment groups (69,222).

(**D**) Genomic heatmap of total reproducible genome-wide CTCF-binding peak signals centered at peak midpoint from RNase A and no treatment groups (38,728).

Additional File 1: Fig. S4. CTCF's RNA binding region deficiency or global RNA depletion plays a limited role in genome-wide chromatin accessibility and transcription.

(**A**) Genomic heatmap of genome-wide ATAC-seq signals centered at reproducible ATAC-seq peak midpoints from $CTCF^{AID2/WT}$ cells and $CTCF^{AID2/dRBR}$ cells (137,540). ATAC-seq tracks from $CTCF^{AID2}$ with or without auxin treatment were shown as controls.

(**B**) Spearman's correlation of all ATAC-seq signals defined by (A) was calculated to quantify the similarity between samples.

(**C**) Chart comparing differential ATAC-seq peaks between dRBR vs WT at different cutoff stringencies.

(**D**) Total RNA-seq was performed to quantify the global gene expression changes between CTCF-WT and CTCF-dRBR groups. Differential expression genes were presented based on different cutoff stringencies. N=2.

(**E**) Total RNA-seq was performed to quantify the global gene expression changes between CTCF-dRBR and CTCF-WT groups based on the cutoff of FDR<0.05. N=2.

(**F**) Total RNA-seq was performed to quantify the global gene expression changes between CTCF-WT and CTCF-dRBR groups. Differential expression genes were presented based on different cutoff stringencies. N=3.

Fig. S1

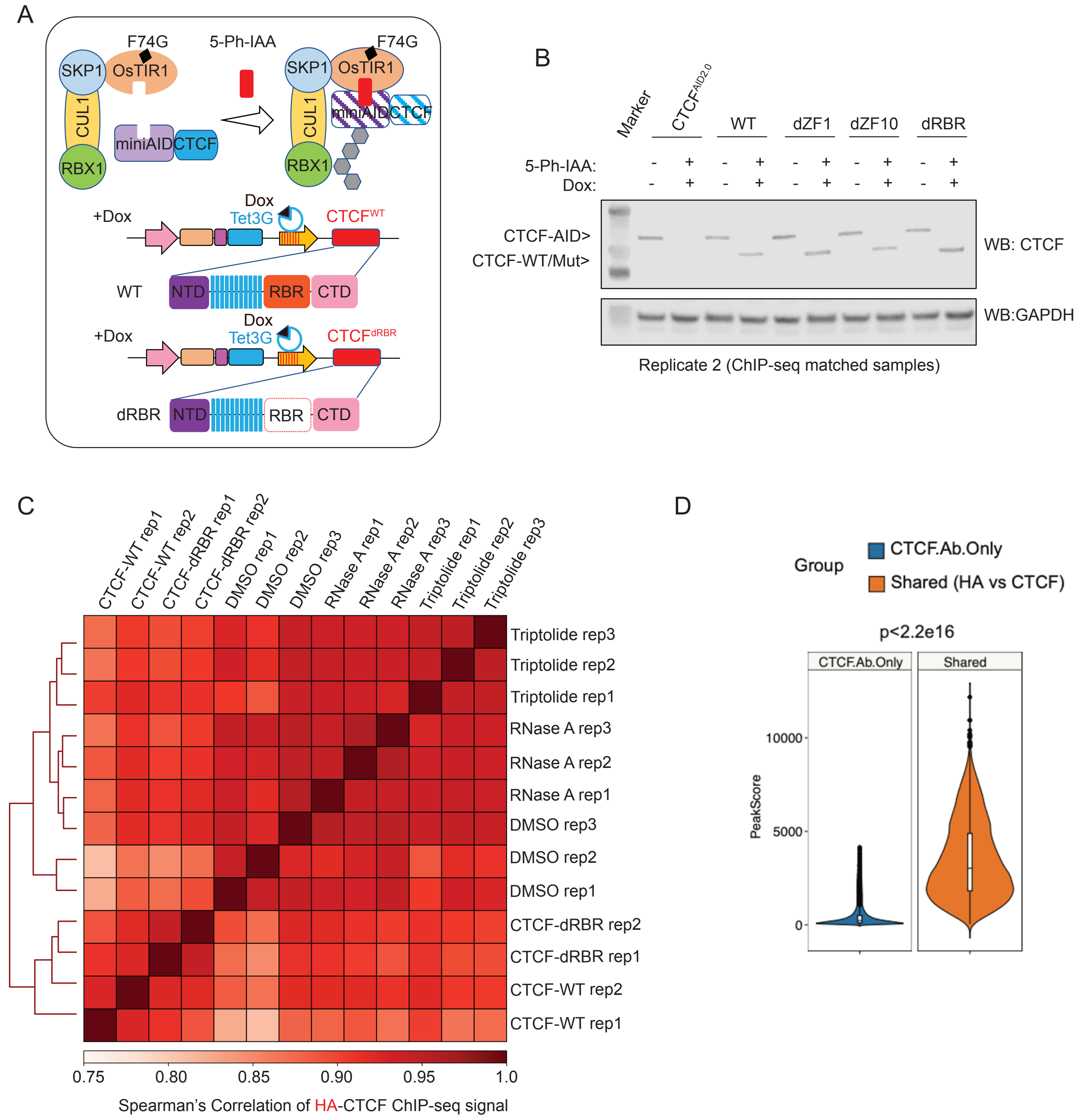
A
F74G
SKP1
OsTIR1
CUL1
RBX1
miniAID
CTCF
5-Ph-IAA
+Dox
Dox
Tet3G
CTCF^WT
WT
NTD
RBR
CTD
CTCF^dRBR
dRBR
B
Marker
CTCF^AID2.0
WT
dZF1
dZF10
dRBR
5-Ph-IAA:
Dox:
CTCF-AID>
CTCF-WT/Mut>
WB: CTCF
WB:GAPDH
Replicate 2 (ChIP-seq matched samples)
C
CTCF-WT rep1
CTCF-WT rep2
CTCF-dRBR rep1
CTCF-dRBR rep2
DMSO rep1
DMSO rep2
DMSO rep3
RNase A rep1
RNase A rep2
RNase A rep3
Triptolide rep1
Triptolide rep2
Triptolide rep3
0.75
0.80
0.85
0.90
0.95
1.0
Spearman's Correlation of HA-CTCF ChIP-seq signal
D
Group
CTCF.Ab.Only
Shared (HA vs CTCF)
p<2.2e16
CTCF.Ab.Only
Shared
PeakScore
10000
5000
0

Fig. S2

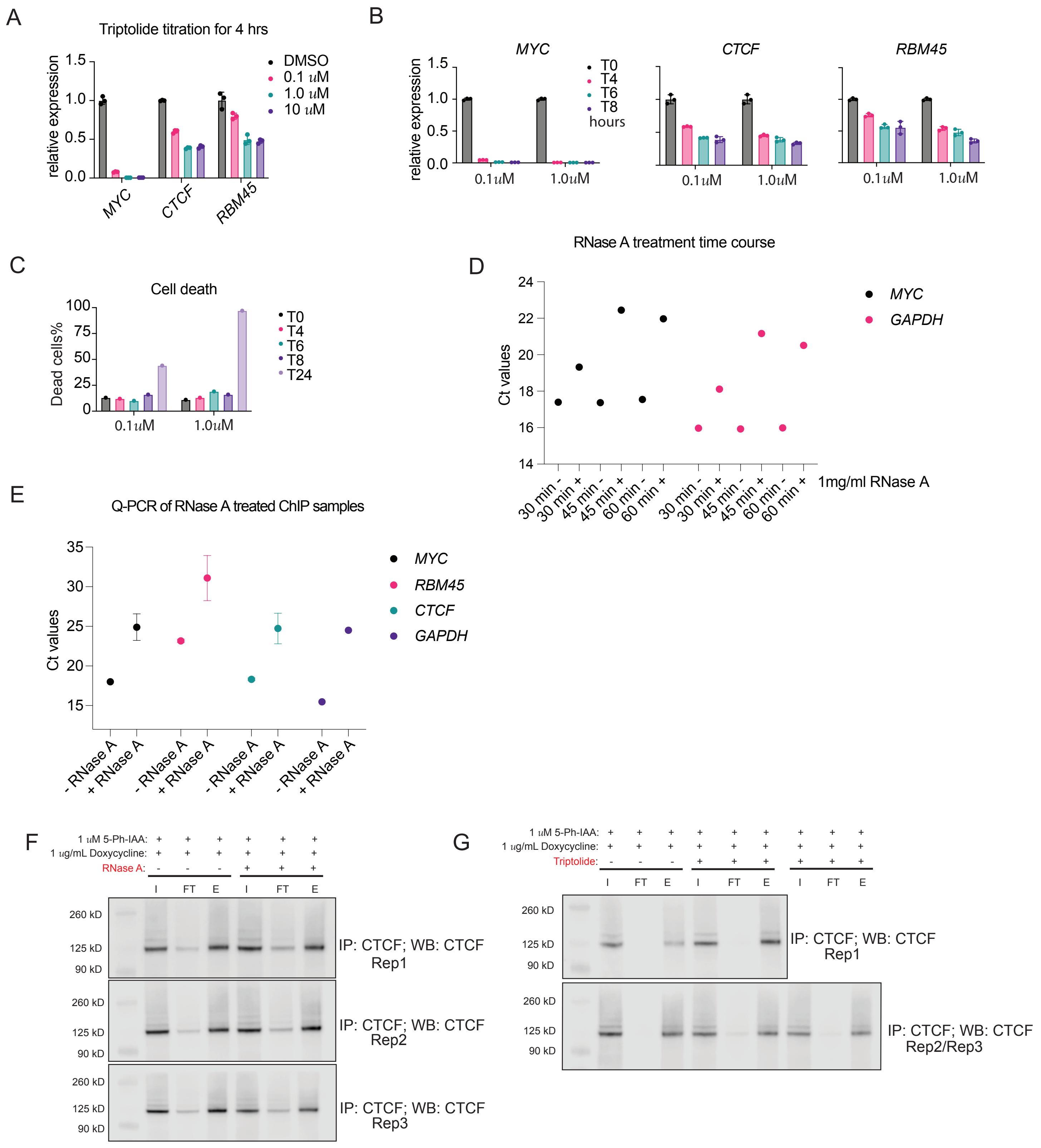
A
Triptolide titration for 4 hrs
relative expression
1.5
1.0
0.5
0.0
DMSO
0.1 uM
1.0 uM
10 uM
MYC
CTCF
RBM45
B
MYC
CTCF
RBM45
T0
T4
T6
T8
hours
0.1uM
1.0uM
C
Cell death
Dead cells%
100
75
50
25
0
T0
T4
T6
T8
T24
0.1uM
1.0uM
D
RNase A treatment time course
Ct values
24
22
20
18
16
14
MYC
GAPDH
30 min -
30 min +
45 min -
45 min +
60 min -
60 min +
1mg/ml RNase A
E
Q-PCR of RNase A treated ChIP samples
Ct values
35
30
25
20
15
MYC
RBM45
CTCF
GAPDH
- RNase A
+ RNase A
F
1 uM 5-Ph-IAA:
1 ug/mL Doxycycline:
RNase A:
I
FT
E
260 kD
125 kD
90 kD
IP: CTCF; WB: CTCF
Rep1
Rep2
Rep3
G
1 uM 5-Ph-IAA:
1 ug/mL Doxycycline:
Triptolide:
IP: CTCF; WB: CTCF
Rep1
Rep2/Rep3

Fig. S3

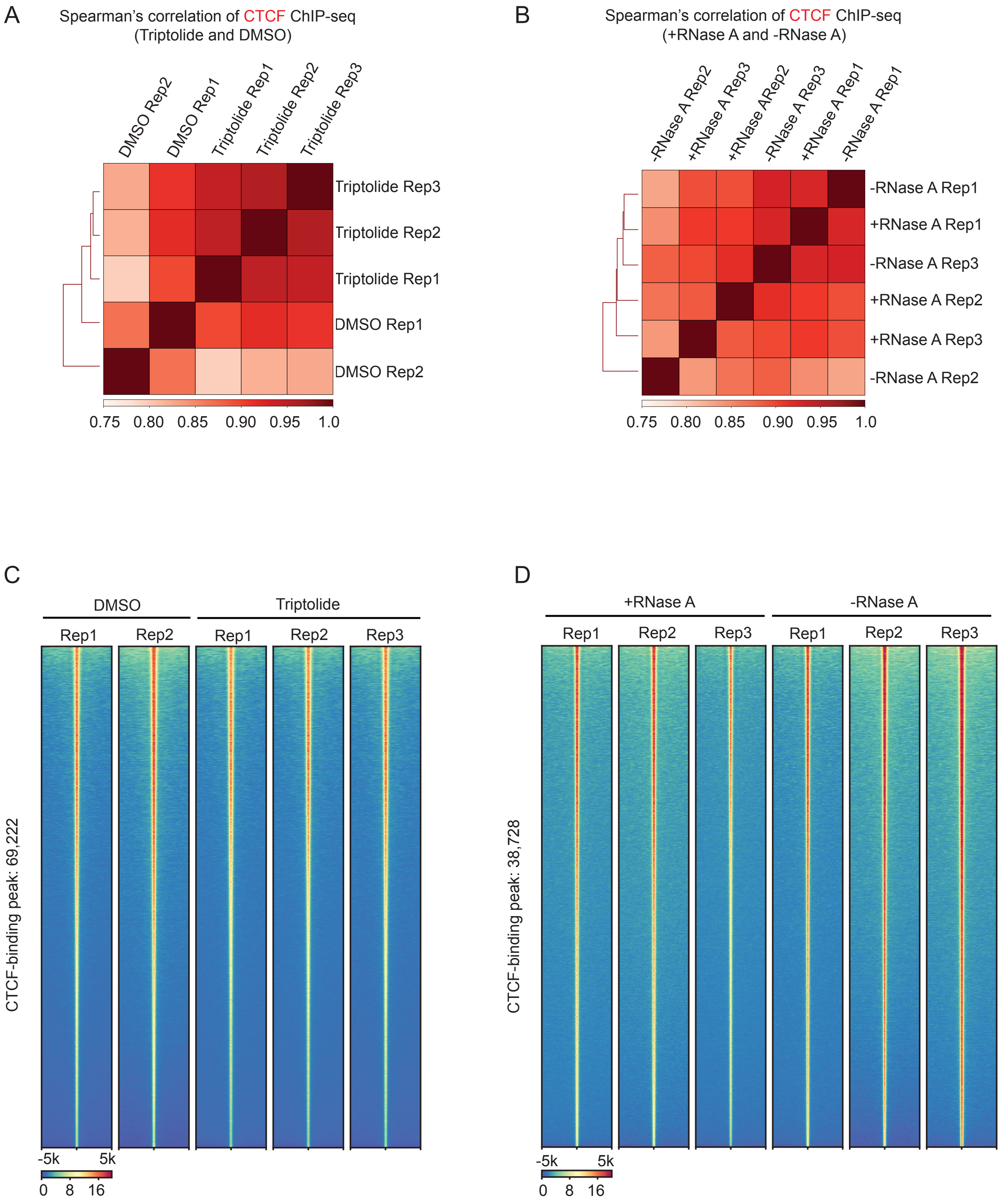
A
Spearman's correlation of CTCF ChIP-seq
(Triptolide and DMSO)
DMSO Rep2
DMSO Rep1
Triptolide Rep1
Triptolide Rep2
Triptolide Rep3
Triptolide Rep3
Triptolide Rep2
Triptolide Rep1
DMSO Rep1
DMSO Rep2
0.75 0.80 0.85 0.90 0.95 1.0
B
Spearman's correlation of CTCF ChIP-seq
(+RNase A and -RNase A)
-RNase A Rep2
+RNase A Rep3
+RNase ARep2
-RNase A Rep3
+RNase A Rep1
-RNase A Rep1
-RNase A Rep1
+RNase A Rep1
-RNase A Rep3
+RNase A Rep2
+RNase A Rep3
-RNase A Rep2
0.75 0.80 0.85 0.90 0.95 1.0
C
DMSO
Triptolide
Rep1
Rep2
Rep1
Rep2
Rep3
CTCF-binding peak: 69,222
-5k
5k
0
8
16
D
+RNase A
-RNase A
Rep1
Rep2
Rep3
Rep1
Rep2
Rep3
CTCF-binding peak: 38,728
-5k
5k
0
8
16

Fig. S4

A

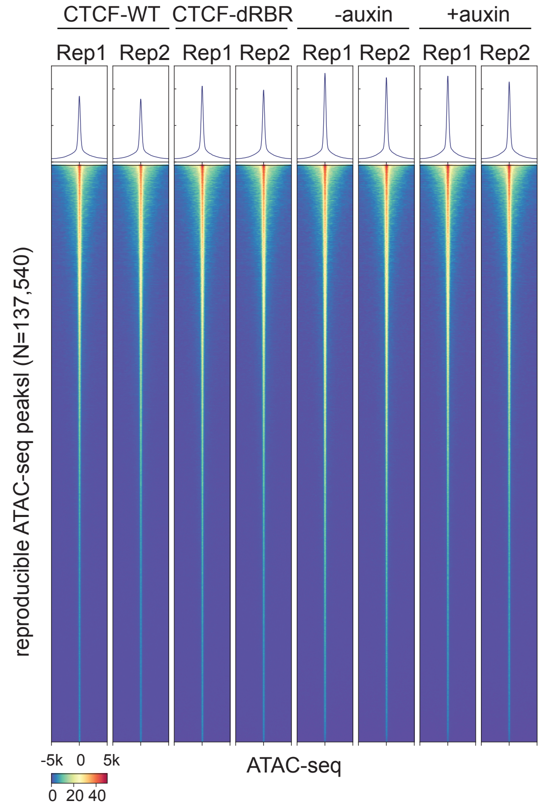

B

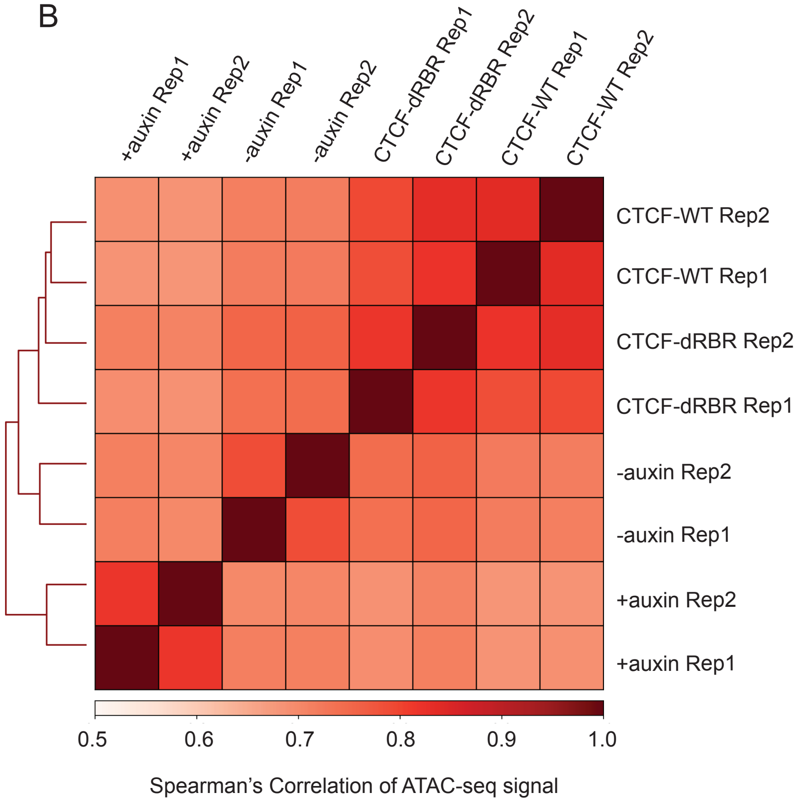

C

Differentially ATAC-seq peaks
(dRBR vs WT)

Cutoff	Up	Down
P value<0.05	9401	7360
FDR<0.05	37	33
FC>2 &P value<0.05	5318	3780
FC>2 & FDR<0.05	3	10

D

Differentially expressed genes
(dRBR vs WT, original batch with 2 replicates each)

Cutoff	Up	Down	Total
P value<0.05	584	625	1209
FDR<0.05	2	1	3
FC>2 & P value<0.05	31	26	57
FC>2 & FDR<0.05	2	1	3

E

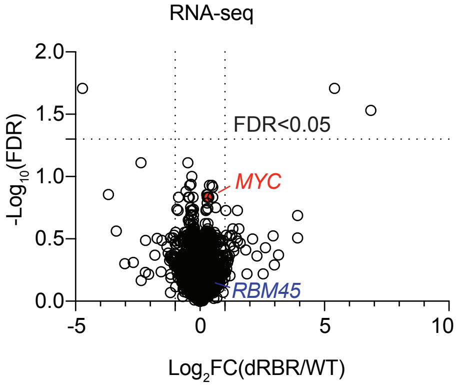

F

Differentially expressed genes
(dRBR vs WT, new batch with 3 replicates each)

Cutoff	Up	Down	Total
P value<0.05	1683	1708	3391
FDR<0.05	0	0	0
FC>2 & P value<0.05	218	389	607
FC>2 & FDR<0.05	0	0	0
