# Supplementary material for: Deciphering the role of RNA in regulating CTCF’s DNA binding affinity in leukemia cells
Source: Genome Biol. 2025 May 12;26:126. doi: 10.1186/s13059-025-03582-x (PMC12067947; doi:10.1186/s13059-025-03582-x)
Supplement: Supplementary file 7 — Additional file 7. Immunoblotting raw data. [file 13059_2025_3582_MOESM7_ESM.pdf]

LI-COR

Image ID: 0000653_02
Acquire Time: Dec 1, 2021 11:12:54 AM

Acquisition Information

#	Image ID	Acquire Time	Channels	Integration Times	Analysis	Image Name	Comment
1	0000653_02	Dec 1, 2021 11:12:54 AM	Chemi	02:05	Manual	0000653_02	

Image Display Values

Channel	Color	Minimum	Maximum	K
Chemi	Gray Scale (Black on White)	0.0000201	0.000703	0.09

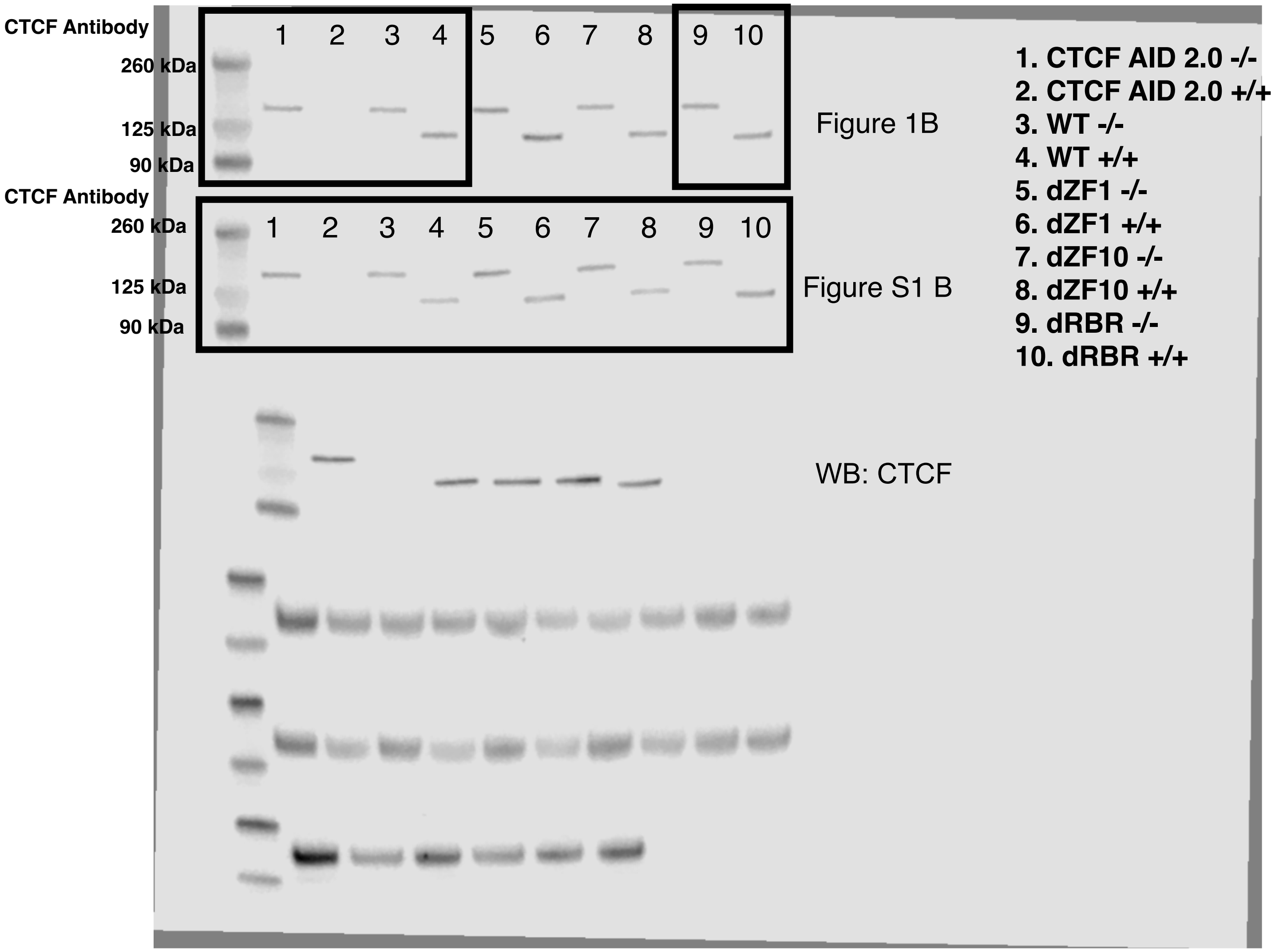

LI-COR

Image ID: 0000654_02
Acquire Time: Dec 1, 2021 11:19:53 AM

Acquisition Information

#	Image ID	Acquire Time	Channels	Integration Times	Analysis	Image Name	Comment
1	0000654_02	Dec 1, 2021 11:19:53 AM	Chemi	02:05	Manual	0000654_02	

Image Display Values

Channel	Color	Minimum	Maximum	K
Chemi	Gray Scale (Black on White)	0.000000775	0.000960	0

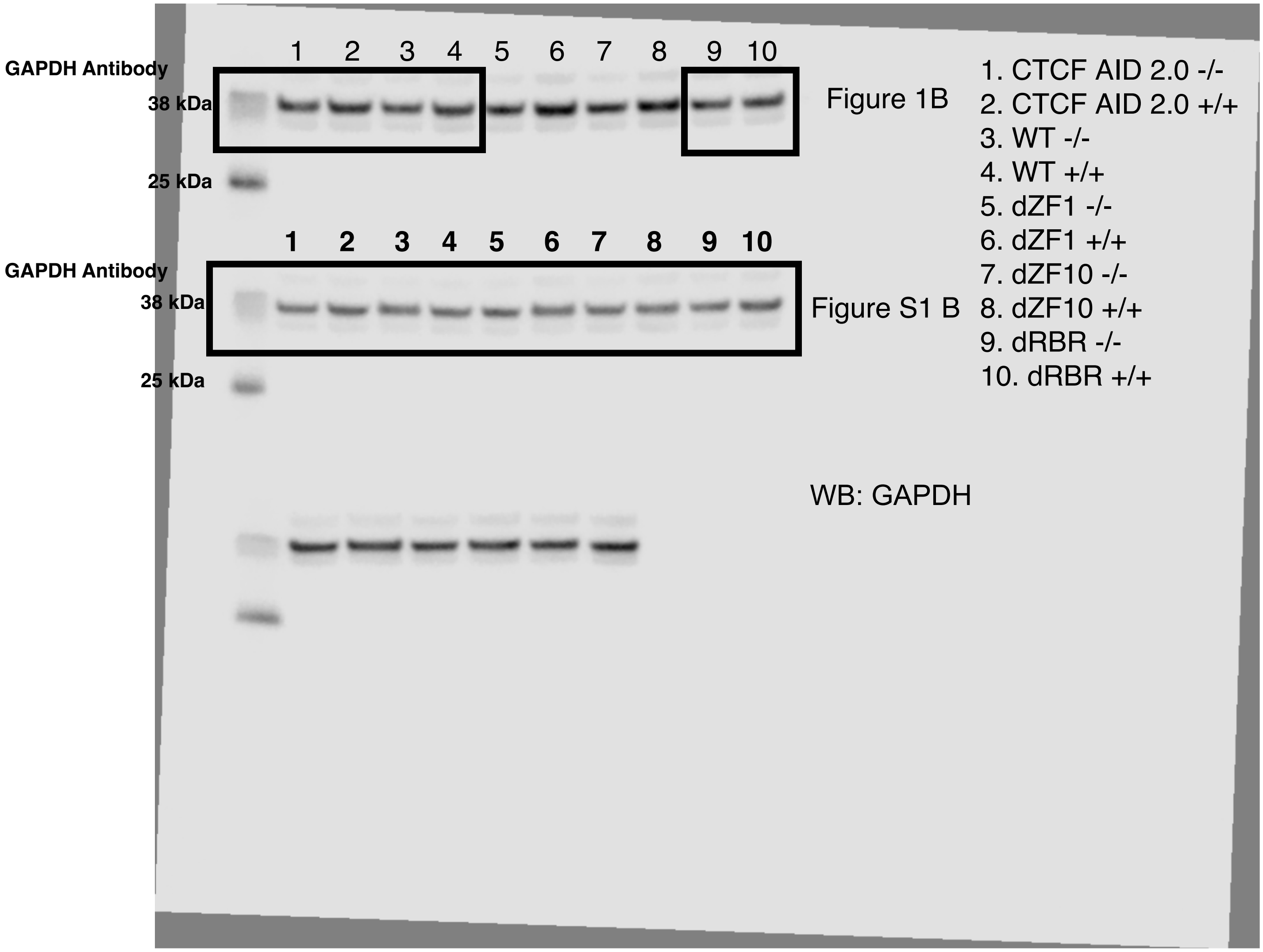

Gel Image

Figure 2 C
QC of RNA seq Library

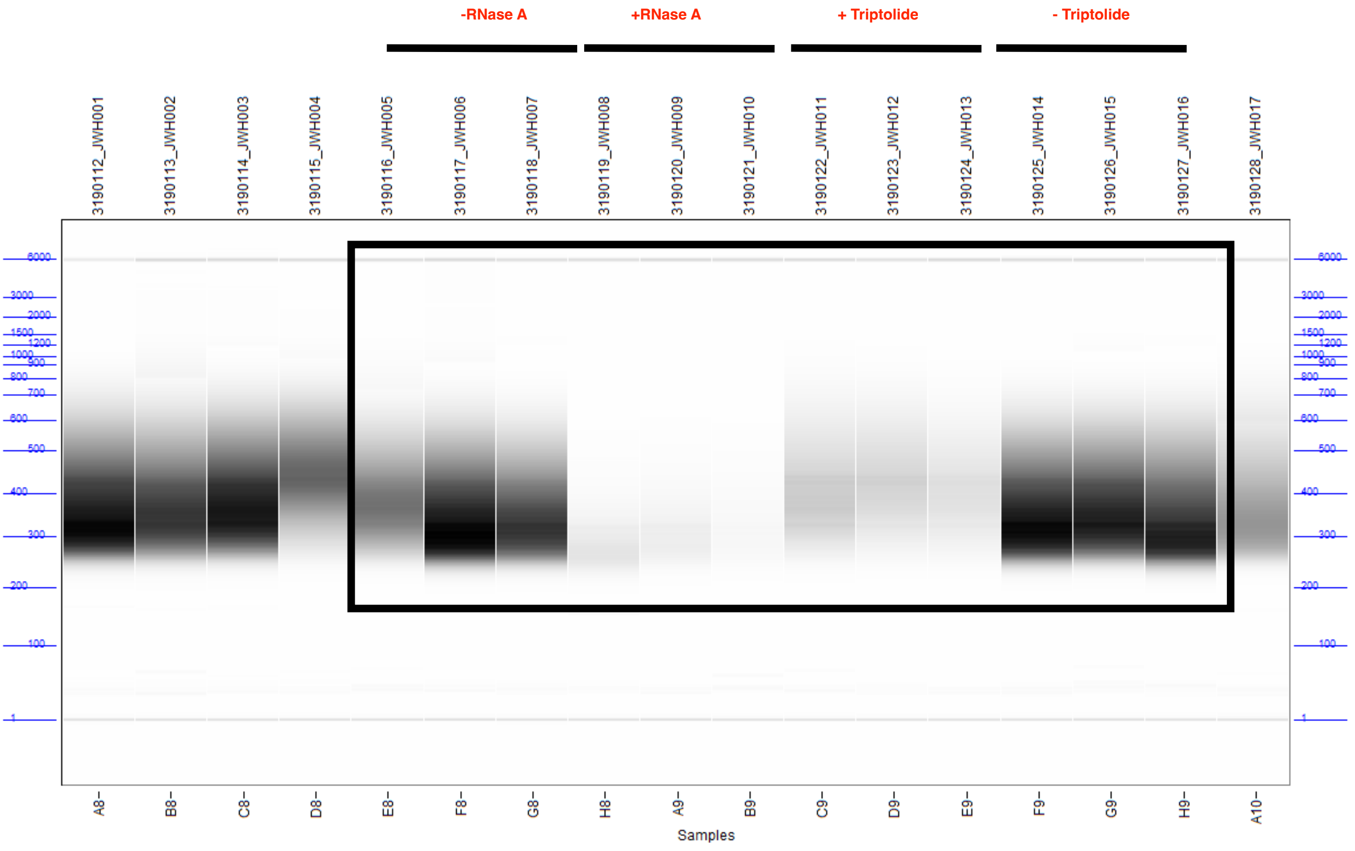

Image ID: 0004365_02
Acquire Time: Sep 19, 2024 10:42:29 AM

Acquisition Information

#	Image ID	Acquire Time	Channels	Integration Times	Analysis	Image Name	Comment
1	0004365_02	Sep 19, 2024 10:42:29 AM	Chemi	02:00	Manual	0004365_02	

Image Display Values

Channel	Color	Minimum	Maximum	K
Chemi	Gray Scale (Black on White)	0.0000121	0.00446	0

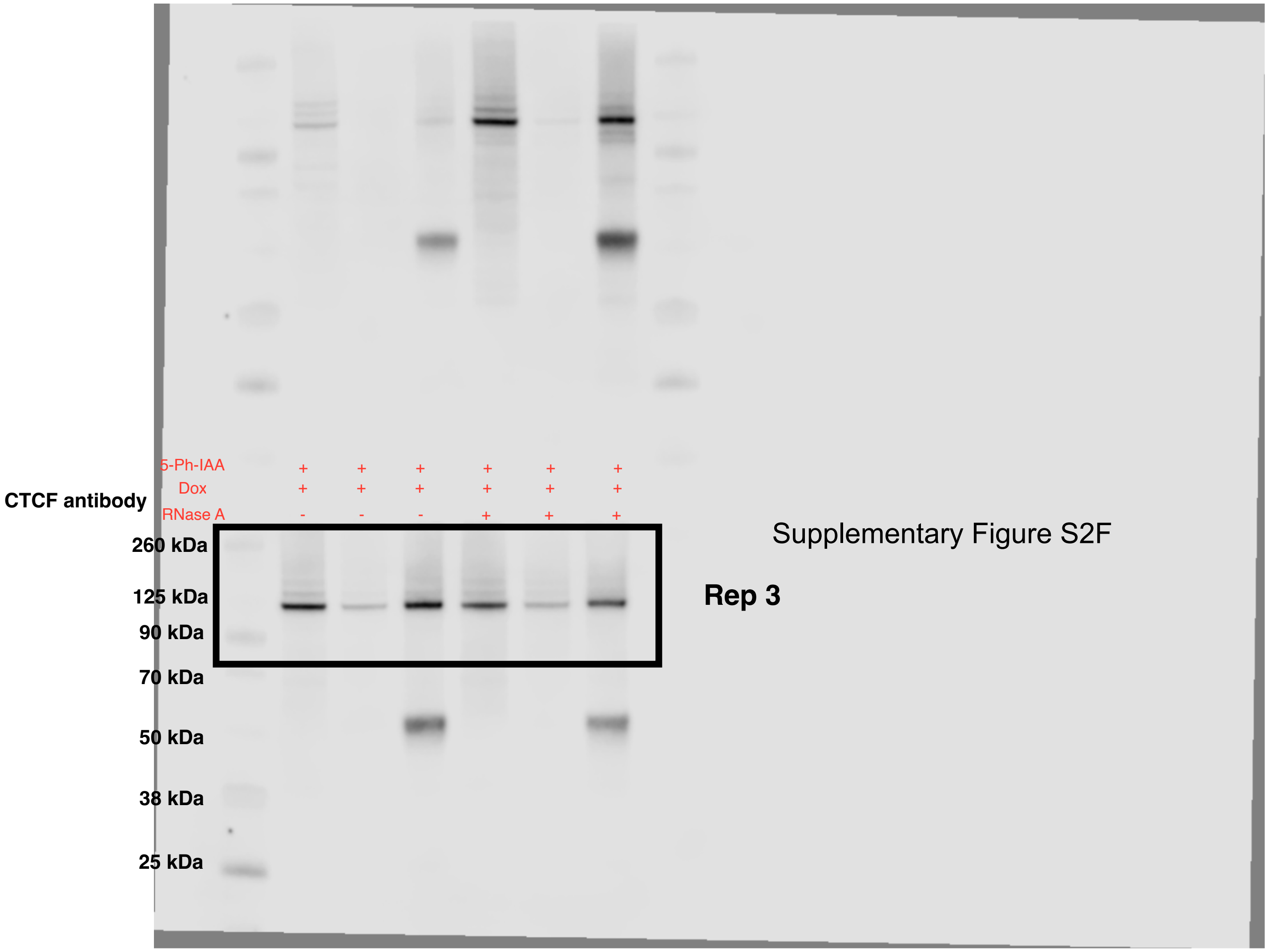

Supplementary Figure S2F

Image ID: 0004347_01
Acquire Time: Sep 13, 2024 9:29:33 AM

Acquisition Information

#	Image ID	Acquire Time	Channels	Integration Times	Analysis	Image Name	Comment	Image Modifications
1	0004347_01	Sep 13, 2024 9:29:33 AM	Chemi	02:00	Manual	0004347_01		

Image Display Values

Channel	Color	Minimum	Maximum	K
Chemi	Gray Scale (Black on White)	0.00000125	0.00322	0.04

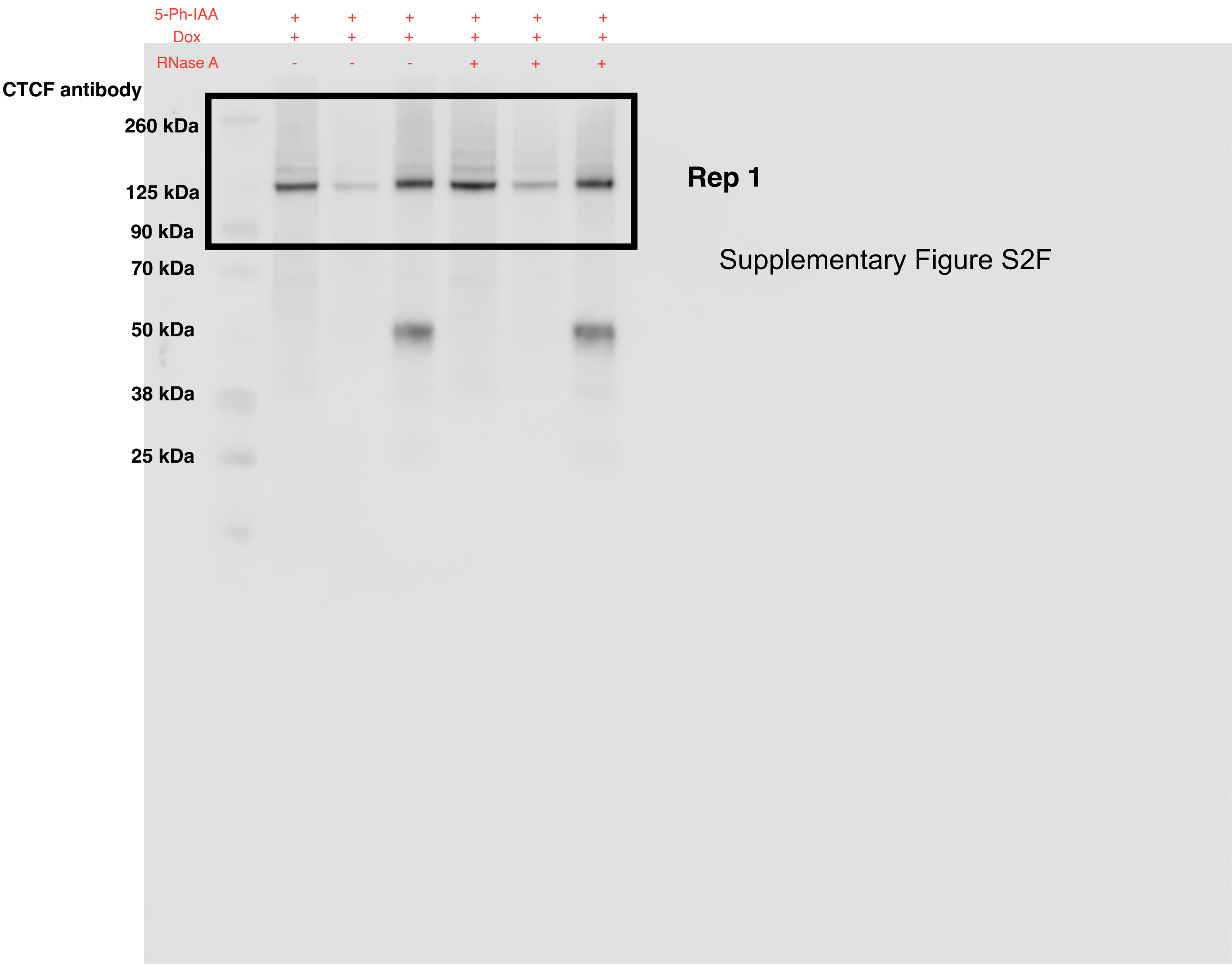

Supplementary Figure S2F

Image ID: 0004361_01
Acquire Time: Sep 18, 2024 9:02:41 AM

Acquisition Information

#	Image ID	Acquire Time	Channels	Integration Times	Analysis	Image Name	Comment	Image Modifications
1	0004361_01	Sep 18, 2024 9:02:41 AM	Chemi	02:00	Manual	0004361_01		

Image Display Values

Channel	Color	Minimum	Maximum	K
Chemi	Gray Scale (Black on White)	0.00000364	0.00299	0

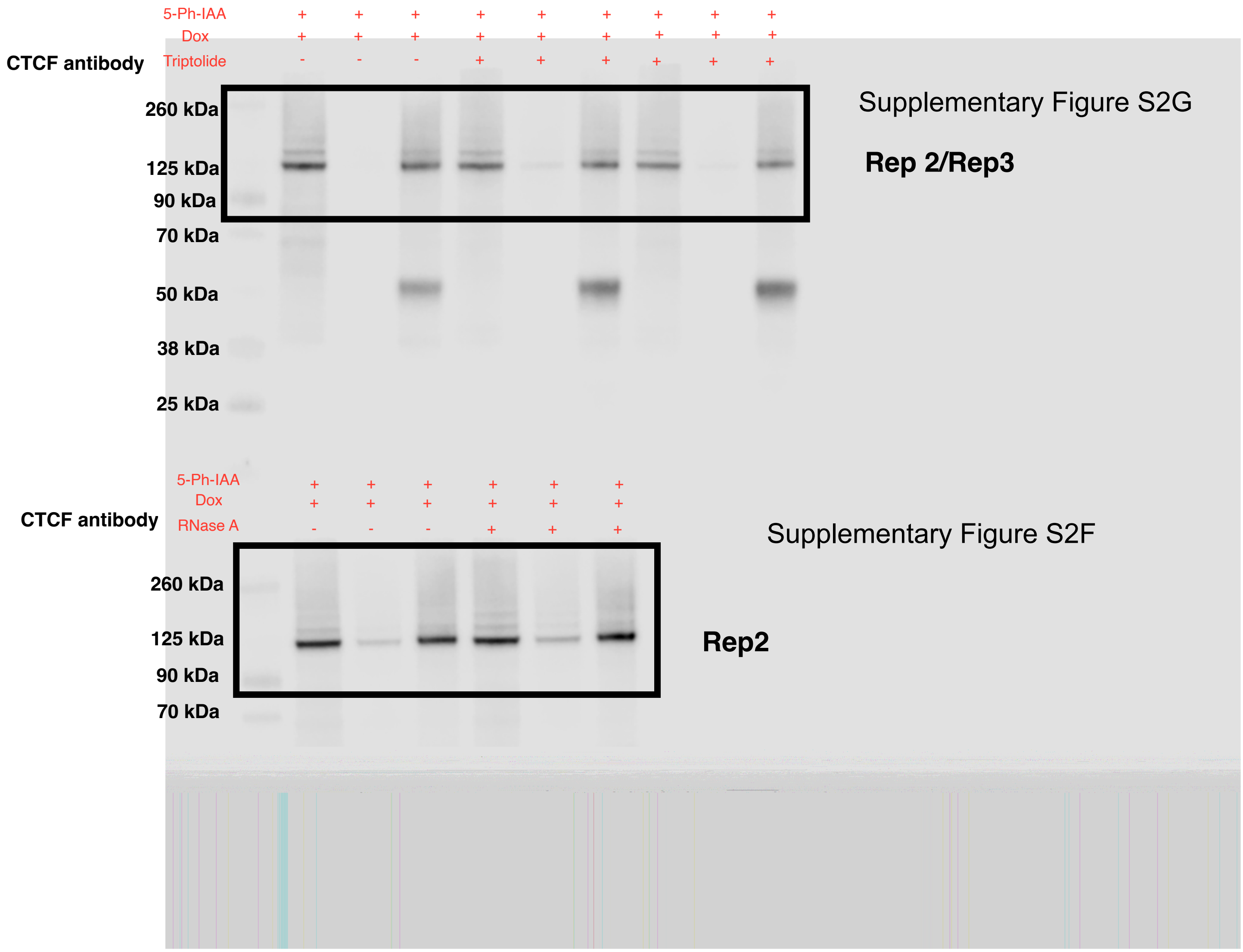

Image ID: 0004346_01
Acquire Time: Sep 13, 2024 9:24:56 AM

Acquisition Information

#	Image ID	Acquire Time	Channels	Integration Times	Analysis	Image Name	Comment	Image Modifications
1	0004346_01	Sep 13, 2024 9:24:56 AM	Chemi	02:00	Manual	0004346_01		

Image Display Values

Channel	Color	Minimum	Maximum	K
Chemi	Gray Scale (Black on White)	0.00000632	0.00248	0

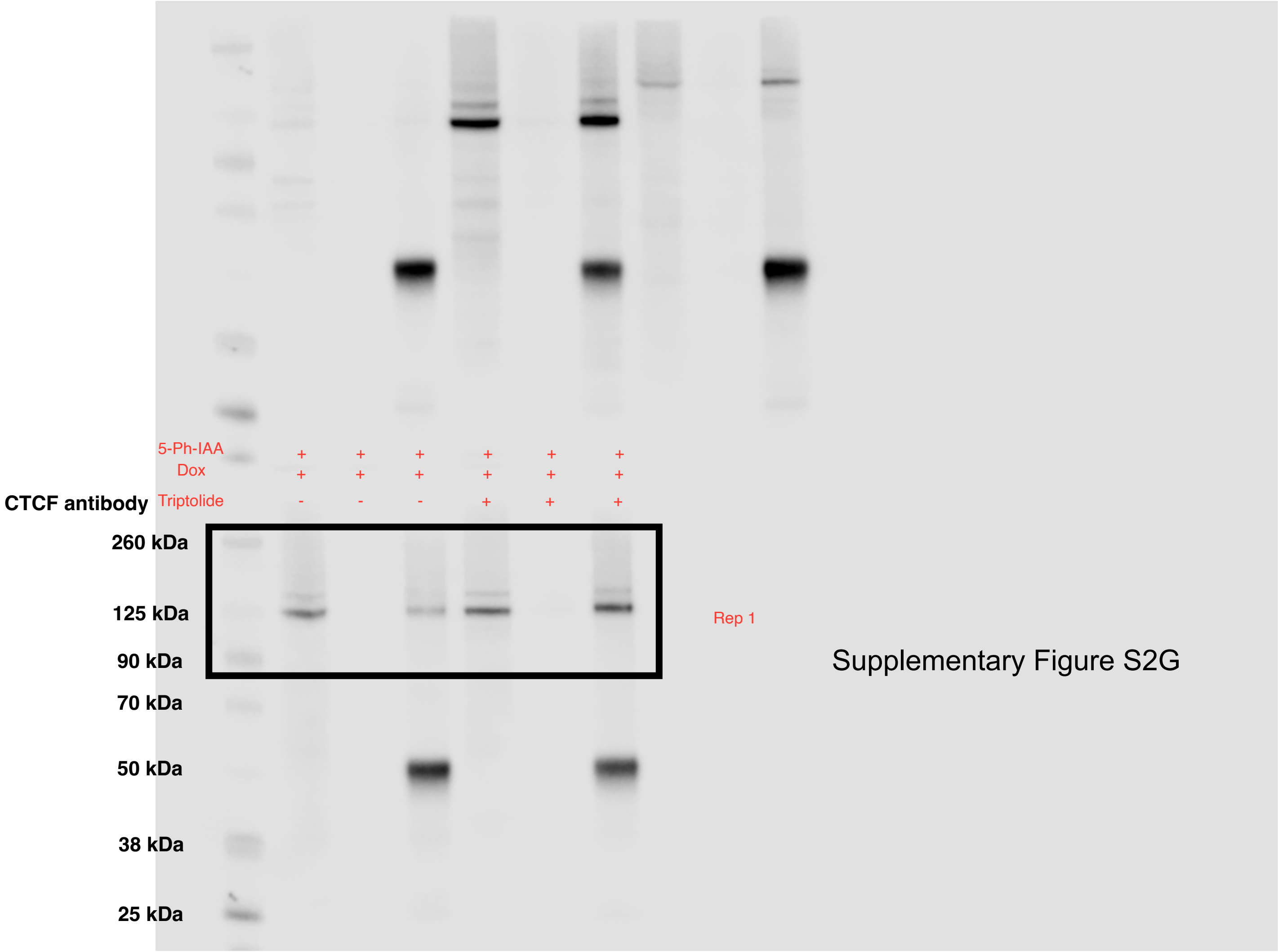

Supplementary Figure S2G
