# Supplementary material for: Deciphering the role of RNA in regulating CTCF’s DNA binding affinity in leukemia cells
Source: Genome Biol. 2025 May 12;26:126. doi: 10.1186/s13059-025-03582-x (PMC12067947; doi:10.1186/s13059-025-03582-x)
Supplement: Supplementary file 8 — Additional file 8. Review history. [file 13059_2025_3582_MOESM8_ESM.docx]

**Review History**

**First round of review**

**Reviewer 1**

**Were you able to assess all statistics in the manuscript, including the appropriateness of statistical tests used?**

 No, I do not feel adequately qualified to assess the statistics.

**Comments to author:**

The paper entitled 'RNA interaction with CTCF plays a limited role in regulating its DNA binding affinity' investigates the potential implications of CTCF-RNA interactions on CTCF's transcriptional regulation ability by three independent approaches that were either deficient for global RNAs or the RNA-binding region of CTCF. By evaluating DNA binding, transcription regulation, and chromatin accessibility, they found that RNA interaction with CTCF plays a limited role in regulating genome-wide chromatin accessibility in a B cell leukemia cell line SEM.

Overall, the finding of this manuscript is interesting. However, there are several statements not supported with data.

Major comments:
As the author claimed 'the possibility of RNA-binding affinity of CTCF may play a role in a tissue-specific manner', the title of this paper could be more focused, such as 'RNA interaction with CTCF plays a limited role in regulating its DNA binding affinity in leukemia cell lines'. Also, the conclusion of this paper need to be verified in primary cells.

The interaction of CTCF and RNA in SEM cells is not clearly shown in the paper.

**Reviewer 2**

**Were you able to assess all statistics in the manuscript, including the appropriateness of statistical tests used?**

Yes, and I have assessed the statistics in my report.

**Comments to author:**

The authors report that RNA interactions play a minor role in CTCF binding in SEM leukemia cells, which contradicts findings from two recent high-profile publications (Saldana-Meyer et al. and Hansen et al. 2019, Mol Cell). I believe this study is not yet ready for publication but may be suitable for Genome Biology after substantial revisions.

The study currently lacks several important quality controls, notably regarding the efficiency of transcription inhibition and RNA degradation. There is also insufficient comparison with the discrepant publications, potentially diminishing its impact on the field.

Below are some suggestions to strengthen the technical aspects of the study and align it with the contrasting 2019 Mol Cell studies. Below are some suggestions to strengthen technical aspects of the study and provide perspective with the contrasting 2019 Mol Cell studies. Double check if Jeannie Lee Jpx papers should be also compared

1. Concerns regarding the efficiency of Pol2 inhibition and RNAse treatment:

To ascertain the effect of Triptolide treatment on nascent transcription, authors report qPCR on MYC and CTCF mRNAS in supp figure 1B. A more comprehensive analysis beyond these two genes is recommended. It is unclear whether normalization to GAPDH is appropriate for quantifying global transcription inhibition. Employing an additional quantitative method to more precisely gauge the GLOBAL level of NASCENT transcription inhibition by Pol2 would be beneficial. Additionally, it would help if the authors clarified whether they used random primers or polydT primers, as these choices impact the type of RNA detected.

The methodology for RNase treatment is not described, making comparisons with prior studies challenging. Please detail how cells/nuclei were collected, permeabilized, treated with RNAseq, and the effectiveness of RNase treatment was determined. Currently, there are no controls verifying the efficiency of RNA degradation after RNase treatment.
after RNAse treatment. Authors need to report the amount of RNA leftover after RNAse digestion.

2. Superficial comparison with previous discrepant studies (points 2, 3 and 4):

Providing more insight into the potential sources of discrepancy with the Saldana-Meyer et al. and Hansen et al. studies would enhance the current study's relevance. Differences in drug treatments, cell lines, genetic approaches, expression levels of mutants, ChIP-seq procedures (including spike-in calibration), replicate numbers, and analytical methods should be discussed. A table summarizing these comparisons would be most beneficial.

3. Spike-in calibration concerns:
I am concerned by the lack of spike-in calibration in the current study could account for the difference with the Saldana-Meyer et al results. It would be informative if authors could reanalyze the Saldana-Meyer data with their pipeline, in two ways: first, using the spike-in normalization. Second, without using the spike-in normalization. If authors notice the difference between control and Triptolide+DRB is only evident with the spike-in normalization approach then it would suggest the calibration approach may account for the discrepancy between studies. Then it would be worth exploring whether the calibration in Saldana-Meyer et al study may have brought artificial sample-to-sample variation that are not necessarily due to the drug treatment. For example, authors may wish to report how many drosophila reads were used by Saladana-Meyer for calibration, and whether these reads might be too low (for example in my experience under 50,000-100,000 reads mapped to the calibrating genome may lead to spurious sample-to-sample various). Again, highlighting the lack of replicates in the Saldana-Meyer study would be insightful to the community.

It is important that authors discuss why they did not resort to using a calibrated ChIP-seq approach using spike-ins, as it sounds like it would have been more appropriate to conclude regarding global occupancy changes. This would have been possible by spiking chromatin from an unrelated HA-tagged line from a different organism (e.g. mouse) that hadn't been subjected to drug treatment. It is not absolutely required that authors repeat all their ChIP-seq experiments using a calibrated approach, as in my direct experience strong global changes are typically still somewhat captured by uncalibrated approach. The fold change is however typically mis-estimated by uncalibrated approaches.

4. Variability in drug treatments and genetic perturbations across studies:

The differences in drug concentrations and treatment durations across studies, including the number of replicates, should be highlighted (again, a comparative table may be ideal). Authors are encouraged to discuss the implications of these differences on their findings.

For example, there are key differences in the drug treatments / genetic perturbations across studies. Saldana-Meyer et al 2019 used 100uM DRB together with 1uM Triptolide) for 4 hour while authors here authors used 10uM for 1h and 4h. They only reported one replicate for each timepoint. Authors used spike-in controls.

Hansen et al Mol Cell 2019 and Nat Chem Biol 2020 deleted the RBRi from endogenous CTCF by replacing it with an HA tag as a linker, in stable knockout cells. They rported reduced CTCF binding overall in the RBRi mutant. Authors used spike ins but reported to not use them for most of their analyses. In this study both 'WT'and 'RBRi' harbor an N-terminal 3xFLAG+Halotag.

Nora et al. 2020 deleted a region largely overlapping with Hansen et al. 2019 but without replacing the deletion by linker. They observed reduced expression of the mutant CTCF transgene and reduced CTCF binding by ChIP-seq compared to WT transgenes, 4 days after induction. Authors did not use spike in normalization in the reported calculations. Authors attributed ChIP-seq and chromosome conformation differences to the reduced expression levels of the RBRi mutant in their experimental set-up.

5. Clarification on ChIP-seq peak selection:

It is important authors clarify how the assembled the list of CTCF peaks used to create figure 1E. What does "reproducible CTCF ChIP-seq peaks"? Is it the list of peaks that overlap across all samples? Or just the DMSO? Ideally authors would need to assemble a list of reproducible CTCF peaks obtained with the CTCF antibody in DMSO treated cells, not considering the other samples. Then the signal obtained with the CTCF antibody in Triptolide, RNAse A, and the signal obtained with the HA antibody from WT or dRBR transgene should be plotted. Also please modify figure key to explain which samples were obtained with CTCF antibody vs HA antibody

6. Expression of different CTCF isoforms in cell lines:

Other studies have reported that uni-terminal tagging of endogenous CTCF in cancer cells (e.g. HAP1) does not deplete all CTCF isoforms, and had to resort to complementing endogenous KO with double-tagged CTCF transgenes (e.g. Valton et al. 2022 NSMB). It would be insightful if authors could provide additional control using various CTCF antibodies, targeting different part of the protein, that all isoforms of endogenous CTCF are fully degraded.

7. It would be important to report that the HA ChIP-seq shows no signal at CTCF peaks when the HA transgenes are not expressed (e.g. using the WT line) [negative control]

Minor details
1. Some figures are unnecessarily busy and confusing:

I think Figure 1B, 1C and 1D not helpful and confusing. For example, hy are cells depicted in fig 1C but not B? What is the point of drawing cells in 1C? Also in 1C what is the red inhibition pointing to RNA supposed to mean? Figure 1D has a type: "Ab beads" not "Ab breads"
I encourage authors to consider simplifying the confusing drawings in 1B, 1C and 1D - or maybe more simply remove them altogether, as they do not add clarity. A line of text just saying "Transcription inhibition with Triptolide", "RNAse treatment", and "HA-CTCF ChIP-seq" is just as helpful, and less confusing that the cartoon drawings.

Figure 1 A - is unnecessary busy, to the point it is very difficult to understand what authors want to highlight. I encourage authors to simplify (e.g. RBX1, CUL1, SKP1 probably do not need to be named beyond 'endogenous ubiquitin machinery') and certainly do not need their own bright color. Many pictograms and colors are not explains (e.g. I assume the gray hexagons with blue outlines are ubiquitin chains? If the point is that CTCF is degraded why not focus on showing that?). Same in the outline of the dox inducible plasmids - I would suggest only identifying the key components to declutter the figure. Finally if the RBR is deleted in the transgene it may be more clear to omit the drawing of the cassette from the dRBR transgene cartoon, instead of just changing background color.

2. As explained in details in Hansen et al and Saldana et al Mol Cell 2019 deletion of the RBRi reduces but does not abrogate CTCF binding to RNA in vitro. IT would be helpful if authors made this more clear in the text.

3. Please clarify how cells expressing WT CTCF of RBR deletion were obtained: please specifically explain if the cell population is polyclonal or if single-clone were derived after infection to ensure homogenous expression of the transgenes.

4. Figure S1E - it may be more informative if authors scaled the color bar to start at the lowest value of the correlation matrix, instead of forcing it to begin at 0. This way small differences in correlation values would become readily visible. Same for S3B and main 2B.

5. Figure S1F it would be clearer if authored marked the diagonal line in black instead of red, given the signal density color scale is mostly red.

6. Line 16, What do authors mean by "Using a gold-standard ChIP-seq assay"? It may be more informative to simply say "ChIP-seq"

7. Saldana et al 2019 reported treated with RNAse BEFORE cross-linking. Here authors report treated AFTER cross-linking. Therefore it needs to be made more clear that conditions cannot be directly compared. If authors really want to contrast with the Saldana-Meyer results they must use the same experimental set up for RNAse treatment.

**Reviewer 3**

**Were you able to assess all statistics in the manuscript, including the appropriateness of statistical tests used?**

Yes, and I have assessed the statistics in my report.

**Comments to author:**

This study examines the role of RNA in the genome-wide binding pattern of CTCF, a DNA binding protein with important functions in 3D genome organization and gene regulation. The authors use 3 different experimental strategies to perturb RNA-CTCF interactions and use ChIP-seq to profile CTCF binding sites genome wide. They do not observe many changes on the 22,091 "reproducible" CTCF binding peaks in several ways of analysis, including CTCF binding level, chromatin accessibility, and gene expression, and conclude that RNA interaction with CTCF does not play a significant role in genome-wide CTCF binding to DNA. The experiments are well designed, and the 3 independent strategies of RNA perturbation is a very comprehensive approach. This study provides valuable data to the community and provides potential insights. However, the data analysis strategy is severely flawed by only focusing on the 22,091 CTCF binding sites, which likely miss the majority of genome-wide CTCF binding sites. Therefore, the conclusion might not be supported by the experimental data due to this biased analysis, making the whole manuscript questionable.

It is well known that CTCF binds to tens of thousands of loci in the human genome. Among those binding sites, around 20k+ binding sites are "constitutive" sites, which are stably conserved across different cell types (Wang et al. PMC3431485). The remaining majority of CTCF binding sites can be cell type specific and play various roles in cell type-specific chromatin structure and gene expression program. It is unsurprising, or even expected, to see that the 20k+ constitutive CTCF binding sites are robust (in both binding level and chromatin accessibility level) against RNA perturbations, as this is consistent with the "constitutive" feature of these sites. The approach that the authors used in identifying the "reproducible" peaks might lose most cell type or condition specific CTCF binding sites, which are exactly those such perturbation experiments should have focused on in the first place. Alternatively, if the merge of all "reproducible" peaks from multiple samples only result in 22,091 peaks, it is possible the ChIP-seq data quality is not good, because a regular working ChIP-seq sample for CTCF should usually contain at least ~50k peaks. How many peaks are there in each sample? It is either the data quality or the flawed analysis strategy (or both) that causes the inability to observe changes in CTCF binding with RNA perturbations.

Because of this significant bias, Figures 1E, 1F, 2A, and 2B are unable to present any real differences as the effect of RNA perturbations. Individual loci such as Figure 1G, 1H, 2C, and 2D are uninformative as well.

As for gene expression, it is an indirect measurement as the possible secondary effect of CTCF binding changes. What are the 3 genes that are identified as significantly differential expression shown in Figure 1I? Are there any CTCF binding changes around these 3 genes? The authors claim that "all of the three gene[s] [were] expressed at a low level (FPKM<1) and were considered as low confident differential expression genes." This is incorrect. Absolute expression level and differential expression are two separate things; one should not disregard the significant differential expression (meeting the FDR cutoff) as "low confident" simply because of their low expression level. They should be experimentally validated. Also, the y-axis label should be negative log FDR.

**Authors Response**

**Point-by-point responses to the reviewers’ comments:**  

Reviewer #1: The paper entitled 'RNA interaction with CTCF plays a limited role in regulating its DNA binding affinity' investigates the potential implications of CTCF-RNA interactions on CTCF's transcriptional regulation ability by three independent approaches that were either deficient for global RNAs or the RNA-binding region of CTCF. By evaluating DNA binding, transcription regulation, and chromatin accessibility, they found that RNA interaction with CTCF plays a limited role in regulating genome-wide chromatin accessibility in a B cell leukemia cell line SEM.

Overall, the finding of this manuscript is interesting. However, there are several statements not supported with data.

Major comments:
As the author claimed 'the possibility of RNA-binding affinity of CTCF may play a role in a tissue-specific manner', the title of this paper could be more focused, such as 'RNA interaction with CTCF plays a limited role in regulating its DNA binding affinity in leukemia cell lines'. Also, the conclusion of this paper need to be verified in primary cells.

*Reply: Thank you for the suggestion. We have updated the title to say, "Deciphering the role of RNA in regulating CTCF’s DNA binding affinity in leukemia cells”. We also appreciate the suggestion to verify the data in primary cells carrying the CTCF-AID knockin. However, as the reviewer may be aware, it is challenging to generate a knockin mouse model for CTCF-AID. Even if it is possible, it will require months of breeding with the OsTIR1-F74G transgenic mouse strain to achieve acute degradation in primary cells. So far as I know, no one has successfully derived this valuable animal tool.*

The interaction of CTCF and RNA in SEM cells is not clearly shown in the paper.

*Reply: Thank you for this comment. RNA has been shown to interact with CTCF in mouse embryonic stem cells (mESCs)(Kung 2015, Saldana-Meyer 2019, Hansen 2019). However, the validity of these in vivo interactions has been debated (Guo 2024, Lee 2024). An improved RNA-IP protocol called covalent linkage and affinity purification (CLAP) showed previous RNA/protein associations observed by crosslinking and immunoprecipitation (CLIP) were largely non-specific and an artifact of the purification conditions. The more stringent CLAP protocol showed CTCF does not interact with RNA in 293T cells. Moreover, data analysis differences also led to opposite conclusions from these studies. As the reported techniques available to decipher true in vivo CTCF/RNA interactions are up to debate, our approach for this study was not to confirm or refute CTCF’s ability to bind RNA, but rather to examine if RNA influences CTCF’s function to bind chromatin. To make this clearer, we updated the language in the paper to define CTCF-RNA interactions as purported interactions.*

Reviewer #2: The authors report that RNA interactions play a minor role in CTCF binding in SEM leukemia cells, which contradicts findings from two recent high-profile publications (Saldana-Meyer et al. and Hansen et al. 2019, Mol Cell). I believe this study is not yet ready for publication but may be suitable for Genome Biology after substantial revisions.

The study currently lacks several important quality controls, notably regarding the efficiency of transcription inhibition and RNA degradation. There is also insufficient comparison with the discrepant publications, potentially diminishing its impact on the field.

Below are some suggestions to strengthen the technical aspects of the study and align it with the contrasting 2019 Mol Cell studies. Below are some suggestions to strengthen technical aspects of the study and provide perspective with the contrasting 2019 Mol Cell studies. Double check if Jeannie Lee Jpx papers should be also compared.

1.      Concerns regarding the efficiency of Pol2 inhibition and RNAse treatment:

To ascertain the effect of Triptolide treatment on nascent transcription, authors report qPCR on MYC and CTCF mRNAS in supp figure 1B. A more comprehensive analysis beyond these two genes is recommended. It is unclear whether normalization to GAPDH is appropriate for quantifying global transcription inhibition. Employing an additional quantitative method to more precisely gauge the GLOBAL level of NASCENT transcription inhibition by Pol2 would be beneficial. Additionally, it would help if the authors clarified whether they used random primers or polydT primers, as these choices impact the type of RNA detected.

*Reply: Thank you for this great comment. To comprehensively justify the Triptolide treatment protocol, we have conducted a systematic quality control (QC) test. For this revision, Triptolide treatment conditions were tested in SEM cells. First, a titration of Triptolide from 0.1 uM to 10 uM was tested over 4 hours. Total RNA was collected, and cDNA was made using random primers. Quantitative PCR (Q-PCR) showed no significant difference in transcript reduction between 1 uM and 10 uM treatments (New Fig. S2A; see below). Next, a time course experiment was carried out treating cells with 0.1 uM or 1 uM Triptolide for 24 hrs. Significant cell death was observed after 24 hrs treatment, as expected. However, neither group showed a significant increase in cell death following 8 hrs of treatment (New Fig. S2B-S2C; see below). Transcription effects were also similar between treatment groups. Since the 1 uM treatment at 8 hrs did not cause an increase in cell death and showed a slight advantage for transcript reduction, this treatment condition was chosen for the study to avoid cell death mediated interference. We collected RNA from CTCF^AID2/WT^ cells at time 0 and 8 hrs after 1 uM triptolide treatment in triplicate that matched the ChIP-seq samples. As suggested by the reviewer, the global steady-state transcript changes following treatment were examined by RNA-extraction and reverse transcription. Library QC showed a marked reduction in* *amplified library cDNA in Triptolide treated cells (New Figure 1C; see below). Therefore, we concluded* *that our Triptolide treatment protocol is a powerful approach to inhibit RNAs in a global manner.*

New Supplementary Figure S2A.

B.

C.

New Supplementary Figure S2B-C.

New Figure 1C. SEM cells treated with DMSO (-) and Triptolide (+) were collected for total RNA extraction and reverse transcription by random-primer oligos. Bioanalyzer analysis was performed to examine the global cDNA levels. Three biological replicate samples were used to ensure reproducibility.

The methodology for RNase treatment is not described, making comparisons with prior studies challenging. Please detail how cells/nuclei were collected, permeabilized, treated with RNAseq, and the effectiveness of RNase treatment was determined.

*Reply: Thank you for this comment. The detailed RNase A treatment protocol for HA-antibody and CTCF-antibody ChIP-seq was provided. A schematic diagram was also shown to describe the whole process (New Figure 2A and Figure 3A; see below). The RNase A ChIP was repeated in triplicate using CTCF^AID2/WT^ cells. Cells were depleted of endogenous CTCF by auxin for 24 hrs. Concurrently, HA tagged CTCF was induced by doxycycline for 18 hrs (starting after 6 hrs of auxin treatment). RNase A treatment conditions were adapted from those previously reported (Saldana-Meyer et al. 2019, Beltran et al. 2016). First, the published treatment conditions were tested for SEM cells. Cells were permeabilized by 0.05% Tween-20 in PBS for 10 minutes on ice. Cells were washed once with PBS and resuspended in PBS plus 1mg/mL RNase A and rotated for 30 minutes at room temperature. Q-PCR was performed to access the transcript reduction of MYC and GAPDH as reflected by increasing Ct values. A modest change in Ct values was observed between untreated and RNase A treated cells. Therefore, we increased the incubation time with RNase A to include a 45 minute and 60-minute incubation followed by Q-PCR (New Supplementary Fig. S2D; see* *below). A 45-minute incubation with 1mg/mL RNase A was determined to be more effective at removing RNA vs 30 minutes. For ChIP, CTCF^AID2/WT^ cells were permeabilized before fixation by 0.05% Tween-20 in PBS for 10 minutes on ice. Cells were washed once with PBS and resuspended in PBS plus 1mg/mL RNase A or mock treated and rotated for 45 minutes at room temperature. Each treatment group (+RNase A, mock) was performed in triplicate. In addition, global steady-state transcript changes following RNase A treatment were examined by RNA-extraction and reverse transcription. Library QC showed a marked reduction in amplified library cDNA in RNase A treated cells (New Figure 1C; see below).*

New Figure 2A.

New Figure 3A.

New Supplementary Figure S2D.

New Figure 1C. SEM cells treated with RNase A (+) and without RNase A (-) were collected for total RNA extraction and reverse transcription by random-primer oligos. Bioanalyzer analysis was performed to examine the global cDNA levels. Three biological replicate samples were used to ensure reproducibility.

Currently, there are no controls verifying the efficiency of RNA degradation after RNase treatment.
after RNAse treatment. Authors need to report the amount of RNA leftover after RNAse digestion.

*Reply: Thank you for the comment. A fraction of each sample prepared for ChIP was collected following the RNase A treatment to collect total RNA. Steady-state transcript changes were examined by Q-PCR to examine the Ct value changes for select genes (New Fig. S2E). The library QC showed a marked reduction amplified library cDNA in RNase A treated cells (New Figure 1C).*

New Supplementary Figure S2E.

2.      Superficial comparison with previous discrepant studies (points 2, 3 and 4):

Providing more insight into the potential sources of discrepancy with the Saldana-Meyer et al. and Hansen et al. studies would enhance the current study's relevance. Differences in drug treatments, cell lines, genetic approaches, expression levels of mutants, ChIP-seq procedures (including spike-in calibration), replicate numbers, and analytical methods should be discussed. A table summarizing these comparisons would be most beneficial.

*Reply: Thank you for this suggestion. Variable experimental conditions were used between different research groups making direct comparisons with previous studies difficult. Our work was not conducted as a direct comparison to previous investigators findings, but rather as an independent investigation to access the impact of RNAs on CTCF’s function in a mammalian leukemia cell line, SEM, utilizing the improved AID2 system to remove endogenous CTCF while exogenously rescuing CTCF with the inducible CTCF-WT or CTCF-dRBR mutant. We and others have shown the AID1 system causes significant cellular toxicity, which clouds data interpretation (Hyle et al. 2023). As the RNA binding region mutant ChIPs performed by Saldana-Meyer et al. 2019 were performed using the AID1 system and exogenous expression of ZF1 and ZF10 mutants, the findings would not be directly relatable to our study using the AID2 system and the RBR mutant. Additionally, the immunoprecipitations were performed using different approaches, and the cell lines were from different host species.*

*Similarly, Hansen et al. 2019 immunoprecipitated endogenously tagged and mutated CTCF from mESCs by a CTCF antibody, whereas we immunoprecipitated exogenous mutant CTCF from SEM cells by anti-HA beads. Please see the table below for details. The current paper (under revision at Genome Biology) is noted as Hyle et al. 2024 and in blue font.*

Data 1 for reviewer.

Data 2 for reviewer.

3.      Spike-in calibration concerns:
I am concerned by the lack of spike-in calibration in the current study could account for the difference with the Saldana-Meyer et al results. It would be informative if authors could reanalyze the Saldana-Meyer data with their pipeline, in two ways: first, using the spike-in normalization. Second, without using the spike-in normalization. If authors notice the difference between control and Triptolide+DRB is only evident with the spike-in normalization approach then it would suggest the calibration approach may account for the discrepancy between studies. Then it would be worth exploring whether the calibration in Saldana-Meyer et al study may have brought artificial sample-to-sample variation that are not necessarily due to the drug treatment. For example, authors may wish to report how many drosophila reads were used by Saladana-Meyer for calibration, and whether these reads might be too low (for example in my experience under 50,000-100,000 reads mapped to the
calibrating genome may lead to spurious sample-to-sample various). Again, highlighting the lack of replicates in the Saldana-Meyer study would be insightful to the community.

It is important that authors discuss why they did not resort to using a calibrated ChIP-seq approach using spike-ins, as it sounds like it would have been more appropriate to conclude regarding global occupancy changes. This would have been possible by spiking chromatin from an unrelated HA-tagged line from a different organism (e.g. mouse) that hadn't been subjected to drug treatment. It is not absolutely required that authors repeat all their ChIP-seq experiments using a calibrated approach, as in my direct experience strong global changes are typically still somewhat captured by uncalibrated approach. The fold change is however typically mis-estimated by uncalibrated approaches.

*Reply: We appreciate the reviewer's insightful comments. As suggested, we reanalyzed the ChIP-seq data from the Saldana-Meyer paper (GSE125595) using both spike-in and non-spike-in normalization methods. Firstly, we mapped the reads to the mouse-drosophila hybrid genome (mm10-dm6) and split the BAM files into mm10-only and dm6-only reads. As shown in Data 3 for reviewer below, the mm10 read QC statistics are satisfactory, despite varying sequencing depths (34M to 172M reads). Notably, there is no strong correlation between sequencing depth and the number of peaks called, both being within acceptable ranges. However, issues arose with the spike-in for 6 out of 9 samples, containing only up to approximately 4,000 spike-in reads (up to 0.01% of total reads). In contrast, the remaining CTCF samples had acceptable spike-in levels (7.8M to nearly 10M drosophila reads, 6%-11% spike-in percentage). This discrepancy raises concerns about potential in silico removal of spike-in reads before GEO submission or procedural differences during the experiment. Importantly, the mm10-only reads and peak calling statistics do not indicate any significant bias related to the spike-in variability, suggesting these issues are not due to biological reasons. Our analysis reveals a strong negative correlation between spike-in percentage and the number of peaks called among the CTCF-Rescue samples, although this correlation is not statistically significant due to the low sample number and lack of replicates (Pearson’s correlation -0.99, p-value=0.0896). The ZF1Δ-rescue sample, which shows the highest spike-in percentage, exhibits relatively lower global signal enrichment normalized to sequencing depth (Data 3A for reviewer) and the lowest number of peaks called among the three CTCF-mutant rescue samples. To address the spike-in read number issue, we applied the ChIPseqSpikeInFree method [PMID: 31566663], which computationally infers* *scaling factors corresponding to spike-in percentages. As shown in Data 3A for reviewer, the ZF1Δ-rescue sample has the lowest scaling factor, indicating a lower signal relative to WT-Rescue and ZF10Δ-rescue samples. These differences are more pronounced when comparing rescue samples to other samples in the dataset (Data 3B-3C for reviewer). Applying the ChIPseqSpikeInFree method to our dataset revealed no significant bias between CTCF-DMSO and CTCF-Trip conditions in the average spike-in scaled scaling factor (Data 3D for reviewer) or in the cumulative distribution plots (Data 3E for reviewer). Although the CTCF-RNase A condition showed a slightly elevated scaling factor, this difference is minimal (0.24) and insignificant compared to the 4.47-point difference observed in K27M studies. Based on these findings, our dataset does not exhibit global signal shifts, but rather numerous focal signal losses. Consequently, we concluded that spike-in normalization is not required in this case. In summary, we believe this comprehensive analysis addresses the reviewer's concerns and provides clarity on the impact of spike-in normalization on our study's findings.*

Data 3 for reviewer.

*In addition, CTCF ChIP seq was conducted for Triptolide and RNase A pre-fixation ChIP (New Figure 3 and New Supplementary Fig. S3). For these ChIP experiments, Drosophila spike in was included for analysis. Data from the CTCF ChIP supported the original findings from the HA ChIP (Previously Figure 1E-F, now Figure 2B-C).*

4.      Variability in drug treatments and genetic perturbations across studies:

The differences in drug concentrations and treatment durations across studies, including the number of replicates, should be highlighted (again, a comparative table may be ideal). Authors are encouraged to discuss the implications of these differences on their findings.

*Reply: Thank you for the comment. Please see the tables above for comparisons across studies (Data 2 for reviewer).*

For example, there are key differences in the drug treatments / genetic perturbations across studies. Saldana-Meyer et al 2019 used 100uM DRB together with 1uM Triptolide) for 4 hour while authors here authors used 10uM for 1h and 4h. They only reported one replicate for each timepoint. Authors used spike-in controls.

*Reply: Thank you for the comment. Treatment conditions were validated specific to cell type. Frist, SEM cells were treated with variable concentrations of Triptolide over 4 hrs to determine a treatment concentration. Q-PCR was performed to show transcription of a few selected target genes (New Supplementary Figs. S2A-S2C). As no change was observed between 1 uM and 10 uM of Triptolide treatment, the higher dose of 10 uM Triptolide was excluded to reduce cytotoxic effects. Next, SEM cells were treated with 0.1 uM and 1 uM Triptolide over a 24 hr time course. Transcription inhibition of a few target genes was assessed by Q-PCR, and cell death was monitored by trypan blue. Cells treated for 8 hrs with 1 uM Triptolide showed transcript inhibition of the selected target genes without increased cytotoxicity. Global steady-state transcript changes following treatment were examined by RNA-extraction and reverse transcription. Library QC showed a marked reduction in amplified library cDNA in Triptolide treated cells (New Figure 1C). Therefore, this treatment condition was selected for the repeated Triptolide CTCF ChIP, which also included spike-in controls (New Figure 3 and New Supplementary Fig. S3).*

Hansen et al Mol Cell 2019 and Nat Chem Biol 2020 deleted the RBRi from endogenous CTCF by replacing it with an HA tag as a linker, in stable knockout cells. They rported reduced CTCF binding overall in the RBRi mutant. Authors used spike ins but reported to not use them for most of their analyses. In this study both 'WT'and 'RBRi' harbor an N-terminal 3xFLAG+Halotag.

Nora et al. 2020 deleted a region largely overlapping with Hansen et al. 2019 but without replacing the deletion by linker. They observed reduced expression of the mutant CTCF transgene and reduced CTCF binding by ChIP-seq compared to WT transgenes, 4 days after induction. Authors did not use spike in normalization in the reported calculations. Authors attributed ChIP-seq and chromosome conformation differences to the reduced expression levels of the RBRi mutant in their experimental set-up.

5.      Clarification on ChIP-seq peak selection:

It is important authors clarify how the assembled the list of CTCF peaks used to create figure 1E. What does "reproducible CTCF ChIP-seq peaks"? Is it the list of peaks that overlap across all samples? Or just the DMSO? Ideally authors would need to assemble a list of reproducible CTCF peaks obtained with the CTCF antibody in DMSO treated cells, not considering the other samples. Then the signal obtained with the CTCF antibody in Triptolide, RNAse A, and the signal obtained with the HA antibody from WT or dRBR transgene should be plotted. Also please modify figure key to explain which samples were obtained with CTCF antibody vs HA antibody

*Reply: Thank you for this comment. Just to clarify, all the ChIPs in primary submission original Figure 1E (now New Figure 2B) were HA-ChIP of exogenously induced CTCF with an HA tag. Endogenous CTCF with the miniAID-mClover tag was depleted by auxin for 24 hrs. After 6 hrs of depletion, doxycycline was added to induce CTCF-WT-HA or CTCF-dRBR-HA concurrently with continued auxin treatment. Since the treatment protocol of each experiment is different, there is an batch effect regarding treatment difference and antibody effect. So reproducible peaks were called within each treatment group. For each sample, both “high confidence peaks” (parameter “-q 0.05”) and “low confidence peaks” (parameter “-q 0.5”) were called. Reproducible peaks (called as a high confidence peak in at least one replicate that also overlapped with a low confidence peak in the other replicates) were generated for each cell type. Peaks that passed the criteria across cell types were combined as reference peaks. For each peak, the number of overlapping ChIP-seq fragments was counted. In the primary submission, only HA-tagged based ChIP-seq were done. In the revision work, we have conducted the ChIP-seq experiments by CTCF antibody in addition to the original HA-tag mediated ChIP-seq. It turns out that CTCF antibody-based ChIP-seq truly identified more binding peaks compared to the HA-tag setting, and our main conclusion remains the same. We provided a summary table here to describe the peak number of each sample and reproducible number in each comparison (Supplementary Table S1).*

Supplementary Table S1.

6.      Expression of different CTCF isoforms in cell lines:

Other studies have reported that uni-terminal tagging of endogenous CTCF in cancer cells (e.g. HAP1) does not deplete all CTCF isoforms, and had to resort to complementing endogenous KO with double-tagged CTCF transgenes (e.g. Valton et al. 2022 NSMB). It would be insightful if authors could provide additional control using various CTCF antibodies, targeting different part of the protein, that all isoforms of endogenous CTCF are fully degraded.

*Reply:*
*Thank you for the comment. We have done isoform analysis at transcript level in our previous study (shown below, from Xu et al. Genome Biology, 2023 Jan 26;24(1):14). As the reviewer suggested, to exclude the possibility that other isoforms of CTCF could be present, we ran Kallisto analysis (Bray, N.L., et al., Near-optimal probabilistic RNA-seq quantification. Nat Biotechnol, 2016. 34(5): p. 525-7.) on total stranded RNA-seq of CTCFAID2 samples with and without 5-Ph-IAA treatment for 1, 4 and 7 days to estimate the genome-wide isoform expression. The abundance of each CTCF isoform was calculated as the transcripts per million mapped reads (TPM) of the isoform divided by the sum of the TPM values of all CTCF isoforms in the same sample. The mean value between replicates was calculated. The top three abundant isoforms of CTCF are shown below. Isoform X-1, which corresponds to the fully transcribed* CTCF, was the most

Data 4 for reviewer.

*dominant isoform and represented in all samples. This is the isoform that was targeted by miniAID. Isoforms X-2 and X-3 were minimally detectable, and no isoforms changed expression patterns following auxin treatment. Moreover, in our unpublished data, we have done IP-Mass Spectrum using CTCF antibody* *to pull down CTCF and its interaction proteins. We only detected one major isoform, which was targeted by miniAID knockin. Therefore, all endogenous CTCF proteins were fully degraded.*

7.      It would be important to report that the HA ChIP-seq shows no signal at CTCF peaks when the HA transgenes are not expressed (e.g. using the WT line) [negative control]

*Reply: Thank you for the comment. We have conducted HA-ChIP-seq against samples with and without doxycycline treatment. About 25,141 and 19,043 peaks were called in TRE3G-WT-CTCF-HA cells with doxycycline treatment. In no doxycycline treatment samples, 588 and 396 peaks were called respectively from two replicates. There are only 104 reproducible peaks between two no Dox samples. Among these 104 peaks, 71 peaks overlap with Dox-treatment samples. CTCF motifs are enriched in the reproducible peaks through the HOMER known motif analysis. Those small number of peaks from no doxycycline groups are likely due to the minimal leakage of the TetOn system. However, these small number of peaks do not affect our major conclusion.*

Minor details
1.      Some figures are unnecessarily busy and confusing:

I think Figure 1B, 1C and 1D not helpful and confusing. For example, why are cells depicted in fig 1C but not B? What is the point of drawing cells in 1C? Also in 1C what is the red inhibition pointing to RNA supposed to mean? Figure 1D has a type: "Ab beads" not "Ab breads"

*Reply: Thank you for this suggestion. We have re-organized the figure representation and updated the text accordingly. We apologize for the typo and carefully revised all text as well.*

I encourage authors to consider simplifying the confusing drawings in 1B, 1C and 1D  - or maybe more simply remove them altogether, as they do not add clarity. A line of text just saying "Transcription inhibition with Triptolide", "RNAse treatment", and "HA-CTCF ChIP-seq" is just as helpful, and less confusing that the cartoon drawings.

Figure 1 A - is unnecessary busy, to the point it is very difficult to understand what authors want to highlight. I encourage authors to simplify (e.g. RBX1, CUL1, SKP1 probably do not need to be named beyond 'endogenous ubiquitin machinery') and certainly do not need their own bright color. Many pictograms and colors are not explains (e.g. I assume the gray hexagons with blue outlines are ubiquitin chains? If the point is that CTCF is degraded why not focus on showing that?). Same in the outline of the dox inducible plasmids - I would suggest only identifying the key components to declutter the figure. Finally if the RBR is deleted in the transgene it may be more clear to omit the drawing of the cassette from the dRBR transgene cartoon, instead of just changing background color.

*Reply: Thank you for this suggestion. We generated a simplified cartoon diagram to illustrate the model system and treatment procedure (New Figure 1A, New Figure 2A and New Figure 3A).*

2.      As explained in details in Hansen et al and Saldana et al Mol Cell 2019 deletion of the RBRi reduces but does not abrogate CTCF binding to RNA in vitro. IT would be helpful if authors made this more clear in the text.

*Reply: Thank you for this suggestion. We have updated the text accordingly.*

3.      Please clarify how cells expressing WT CTCF of RBR deletion were obtained: please specifically explain if the cell population is polyclonal or if single-clone were derived after infection to ensure homogenous expression of the transgenes.

*Reply: Thank you for this comment. The SEM B-ALL cell lines CTCF^AID2^, CTCF^AID2/WT,^ and CTCF^AID2/dRBR^ were previously described (Hyle et al. 2023). In brief, CTCF^AID2^ cells have an in-frame miniAID-mClover3 tag at the C-terminus of endogenous CTCF and constitutively express OsTIR1 F74G. The CTCF^AID2/WT^ and CTCF^AID2/dRBR^ cells were CTCF^AID2^ cells transduced with the doxycycline-inducible wild-type HA-tagged CTCF and the HA-tagged CTCF-dRBR mutant, respectively. They are bulk populations for ectopic expression of transgenes. More importantly, upon doxycycline induction, the expression level of CTCF wildtype and dRBR mutant are the same (New Figure 1B).*

New Figure 1B.

4.      Figure S1E - it may be more informative if authors scaled the color bar to start at the lowest value of the correlation matrix, instead of forcing it to begin at 0. This way small differences in correlation values would become readily visible. Same for S3B and main 2B.

*Reply: Thank you for this suggestion. We have made the changes accordingly as requested. The figure locations are now New Figure 4B, New Supplementary Figs. S1C, S3A and S3B.*

5.      Figure S1F it would be clearer if authored marked the diagonal line in black instead of red, given the signal density color scale is mostly red.

*Reply: Thank you for this suggestion. This supplementary figure is not included in the revision.*

6.      Line 16, What do authors mean by "Using a gold-standard ChIP-seq assay"? It may be more informative to simply say "ChIP-seq"

*Reply: Thank you for this suggestion. We have updated the text accordingly.*

7.      Saldana et al 2019 reported treated with RNAse BEFORE cross-linking. Here authors report treated AFTER cross-linking. Therefore it needs to be made more clear that conditions cannot be directly compared. If authors really want to contrast with the Saldana-Meyer results they must use the same experimental set up for RNAse treatment.

*Reply: We agree with your suggestion. RNase A treated ChIP seq experiments were repeated to remove RNAs prior to fixation. New data are shown in New Figure 3 and New Supplementary Fig. S3.*

New Figure 3A.

Reviewer #3: This study examines the role of RNA in the genome-wide binding pattern of CTCF, a DNA binding protein with important functions in 3D genome organization and gene regulation. The authors use 3 different experimental strategies to perturb RNA-CTCF interactions and use ChIP-seq to profile CTCF binding sites genome wide. They do not observe many changes on the 22,091 "reproducible" CTCF binding peaks in several ways of analysis, including CTCF binding level, chromatin accessibility, and gene expression, and conclude that RNA interaction with CTCF does not play a significant role in genome-wide CTCF binding to DNA. The experiments are well designed, and the 3 independent strategies of RNA perturbation is a very comprehensive approach. This study provides valuable data to the community and provides potential insights. However, the data analysis strategy is severely flawed by only focusing on the 22,091 CTCF binding sites, which likely miss the majority of genome-wide

CTCF binding sites. Therefore, the conclusion might not be supported by the experimental data due to this biased analysis, making the whole manuscript questionable.

It is well known that CTCF binds to tens of thousands of loci in the human genome. Among those binding sites, around 20k+ binding sites are "constitutive" sites, which are stably conserved across different cell types (Wang et al. PMC3431485). The remaining majority of CTCF binding sites can be cell type specific and play various roles in cell type-specific chromatin structure and gene expression program. It is unsurprising, or even expected, to see that the 20k+ constitutive CTCF binding sites are robust (in both binding level and chromatin accessibility level) against RNA perturbations, as this is consistent with the "constitutive" feature of these sites. The approach that the authors used in identifying the "reproducible" peaks might lose most cell type or condition specific CTCF binding sites, which are exactly those such perturbation experiments should have focused on in the first place. Alternatively, if the merge of all "reproducible" peaks from multiple samples only result in 22,091 peaks, it is possible the ChIP-seq data quality is not good, because a regular working ChIP-seq sample for CTCF should usually contain at least ~50k peaks. How many peaks are there in each sample? It is either the data quality or the flawed analysis strategy (or both) that causes the inability to observe changes in CTCF binding with RNA perturbations. Because of this significant bias, Figures 1E, 1F, 2A, and 2B are unable to present any real differences as the effect of RNA perturbations. Individual loci such as Figure 1G, 1H, 2C, and 2D are uninformative as well.

*Reply: We appreciate the provided positive comments and insightful critiques. We have conducted the ChIP-seq experiments by CTCF antibody in addition to HA-tag mediated ChIP-seq. It turns out that CTCF antibody-based ChIP-seq truly identified more binding peaks compared to the HA-tag setting, and our main conclusion remains the same (New Figure 3). We also optimized our RNase A treatment protocol by adding the enzyme prior to fixation procedure as requested by the reviewers, and we obtained the similar result that RNA interaction with CTCF plays a limited role in regulating its DNA binding affinity in a global manner.*

New Figure 3A.

New Supplementary Fig. S1D

*According to the reviewers’ comment, we compared the peakscore of CTCF-antibody only peaks (New Supplementary Fig. S1D: CTCF Ab Only) and shared peaks between HA and CTCF-pulldown (New Supplementary Fig. S1D: Shared). It turns out HA-ChIP-seq pulled down stronger CTCF binding chromatins. Therefore, we repeated the RNase A and Triptolide treatment experiment and performed ChIP-seq using CTCF antibody. CTCF-antibody mediated ChIP-seq indeed identified much more peaks compared with HA-tag-mediated ChIP-seq and we obtained the similar result to support that RNA interaction with CTCF plays a limited role in regulating its DNA binding affinity (New Figure 3, New Supplementary Fig. S3).*

As for gene expression, it is an indirect measurement as the possible secondary effect of CTCF binding changes. What are the 3 genes that are identified as significantly differential expression shown in Figure 1I? Are there any CTCF binding changes around these 3 genes? The authors claim that "all of the three gene[s] [were] expressed at a low level (FPKM<1) and were considered as low confident differential expression genes." This is incorrect. Absolute expression level and differential expression are two separate things; one should not disregard the significant differential expression (meeting the FDR cutoff) as "low confident" simply because of their low expression level. They should be experimentally validated. Also, the y-axis label should be negative log FDR.

*Reply: We appreciate the provided insightful critiques. We agree with the reviewer on the difference between absolute expression level and differential expression. Below, we provide the table to describe the top three differential expression genes along with ChIP-seq tracks (Data 5 for the reviewer, Data 6 for the reviewer). Although the three genes passed the FDR test, we failed to identify the correlation between CTCF-binding affinity and gene expression. We also changed the typo of negative LogFDR.*

Data 5 for reviewer. reviewer.

Data 6 for reviewer. reviewer.

**Second round of review**

**Reviewer 1**

I find that the authors have put considerable effort into addressing the reviewers' comments. As a result, the paper is very much improved and I have no problem in it for publication.

**Reviewer 2**

The authors have addressed my concerns with their extensive revisions.

In their rebuttal, the authors present significant re-analyses of previous papers that had proposed a role for RNA in CTCF functions. These re-analyses highlight important weaknesses in the earlier datasets, which I believe are worth including in the supplementary figures of the current manuscript. In particular, it would greatly benefit the field to incorporate the careful inter-study comparisons presented in the rebuttal as ‘Data 1, Data 2, and Data 3 for the reviewer.’ Additionally, an abbreviated description of the rebuttal to reviewer #2's points 2 and 3 could also be included.

Finally, it is essential for the authors to publish annotated plasmid sequences (e.g., GenBank files) for their key constructs, such as the CTCF-AID2 targeting construct, the Tir1 expression construct, the base vector for CTCF cDNA expression, Cas9 vectors, and others. These should be made available either through Addgene or as supplementary data attached to the manuscript.

**Reviewer 3**

The authors partially addressed my comments, and the manuscript has been significantly improved. However, I still have some concerns:

1. As mentioned in the comments to the previous version, the "reproducible" CTCF binding sites are the strongest and constitutive CTCF binding sites, they are not expected to exhibit significant changes after any perturbation. And the highest signals dominate the correlation analysis and the heatmap visualization, which are not the best way to present the data. Instead, I suggest that the authors perform differential ChIP-seq analysis using tools like MAnorm2 or Diffbind to identify differential peaks. The current description of differential analysis is vague and lacks important parameter information. After all, the changes in CTCF weak peaks can still be significant and functionally important, and they should not have been neglected or underestimated.

2. Gene expression data shown in Data 5 for reviewer only have 2 replicates. This is inconsistent with the authors' claim that the experiments were done in 3 replicates. The authors should clarify. 3 replicates for gene expression profiling should be the norm in the field.

3. Data 6 for reviewer only show the CTCF binding patterns in a narrow window (10kb-50kb) around the gene. As CTCF can induce loops and 3D chromatin changes in a larger scale, the authors should examine at least 100-200kb regions.

**Authors Response**

**Point-by-point responses to the reviewers’ comments:**  

Reviewer #1: I find that the authors have put considerable effort into addressing the reviewers' comments. As a result, the paper is very much improved and I have no problem in it for publication.

*Response: Thank you for your positive comments.*

Reviewer #2:
The authors have addressed my concerns with their extensive revisions.

In their rebuttal, the authors present significant re-analyses of previous papers that had proposed a role for RNA in CTCF functions. These re-analyses highlight important weaknesses in the earlier datasets, which I believe are worth including in the supplementary figures of the current manuscript. In particular, it would greatly benefit the field to incorporate the careful inter-study comparisons presented in the rebuttal as ‘Data 1, Data 2, and Data 3 for the reviewer.’ Additionally, an abbreviated description of the rebuttal to reviewer #2's points 2 and 3 could also be included.

*Response: Thank you for your suggestions. We have now included ‘Data 1 and Data 2 for the reviewer’ in Supplementary Table 4. In addition, the discussion has been updated as requested. Please see below. Previous studies by Saldana-Meyer et al. 2019 and Hansen et al. 2019 showed RNAs were essential to CTCF-mediated genome organization. Saldana-Meyer et al. 2019 utilized the auxin-inducible degradation system to degrade endogenous CTCF in mESCs while exogenously expressing WT CTCF and mutants of the putative RNA binding regions, ΔZF1 and ΔZF10. PAR-CLIP showed reduced RNA binding to CTCF in cells expressing ΔZF1 and ΔZF10. Differential chromatin binding, gene expression, and chromatin looping were observed in both mutants compared to cells expressing WT CTCF. RNA was disrupted by triptolide and RNase A in mESCs and showed modest changes to global CTCF chromatin occupancy. However, for all ChIP seq experiments spike-in normalization was variable among samples. Of note, the auxin degradation system available at the time of the study was the original AID1 design, which requires high amounts of auxin to achieve protein degradation. High auxin concentrations have since been shown to cause significant cellular toxicity that could cloud data interpretation (Hyle 2023, Yesbolatova 2020). Hansen et al. 2019 replaced the RBR of CTCF in mESCs with a linker and 3xHA tag to generate an endogenous RBR mutant, ΔRBR_i_-CTCF. ChIP seq using spike-in normalization revealed a global decrease in CTCF chromatin occupancy and disrupted chromatin looping of a set of sites designated to be dependent on the RBR. The authors noted that the protein stability of the ΔRBR_i_-CTCF protein was compromised compared to WT and questioned whether the reduced expression resulted in fewer chromatin contacts. Additionally, ΔRBR_i_-CTCF cells doubling time was greater when compared to WT-CTCF cells. In contrast, we observed equal CTCF protein expression between CTCF^AID2/WT^ and CTCF^AID2/dRBR^ cell lines, and there were no changes to cellular fitness. While these studies provide important insights into the significance of CTCF-RNA interactions, the studies were limited to mESCs and used varied methodologies and analyses, making direct comparisons to our study challenging*

*(Supplementary Table S4).*

Finally, it is essential for the authors to publish annotated plasmid sequences (e.g., GenBank files) for their key constructs, such as the CTCF-AID2 targeting construct, the Tir1 expression construct, the base vector for CTCF cDNA expression, Cas9 vectors, and others. These should be made available either through Addgene or as supplementary data attached to the manuscript.

*Response: Thanks for the suggestion. We have submitted the key plasmids used in this study to Addgene. Enclosed please find the plasmid information and Addgene ID for purchase purposes.*

| **Addgene ID** | **Plasmid** |
| --- | --- |
| 232802 | [TRE3G-CTCF-dRBR-HA-MND-zeo](https://www.addgene.org/232802/) |
| 232801 | [TRE3G-CTCF-WT-HA-MND-zeo](https://www.addgene.org/232801/) |
| 232800 | [pCDH-MND-OsTIR1(F74G)-P2A-EGFP-AID2-EF1a-RFP](https://www.addgene.org/232800/) |

Reviewer #3: The authors partially addressed my comments, and the manuscript has been significantly improved. However, I still have some concerns:

1. As mentioned in the comments to the previous version, the "reproducible" CTCF binding sites are the strongest and constitutive CTCF binding sites, they are not expected to exhibit significant changes after any perturbation. And the highest signals dominate the correlation analysis and the heatmap visualization, which are not the best way to present the data. Instead, I suggest that the authors perform differential ChIP-seq analysis using tools like MAnorm2 or Diffbind to identify differential peaks. The current description of differential analysis is vague and lacks important parameter information. After all, the changes in CTCF weak peaks can still be significant and functionally important, and they should not have been neglected or underestimated.

*Response: We thank the reviewer for proposing different methods of detecting differential peaks. We evaluated how disrupting RNA/CTCF interactions would affect CTCF DNA binding by ChIP-seq using three strategies. Two approaches disrupted RNA by RNase A or Triptolide treatment and a third approach compared CTCF binding between an RBR deficient CTCF mutant and WT CTCF. We also had complementary RNA-seq comparing differential expression between CTCF-dRBR and CTCF-WT cells. The new RNA-seq dataset using three replicates did not find any significant dysregulated genes that passed the FDR 5% cutoff, suggesting detecting more differential peaks between CTCF-dRBR/CTCF-WT cells would not change our conclusion that no or few genes were significantly regulated by CTCF-dRBR compared to wildtype. We did report a small reproducible subset of CTCF binding regions that were shown to have differential binding in both RNase A and Triptolide treated cells (Fig. 3B). However, correlation to transcriptional regulation is not possible in RNase A and Triptolide treated cells.*

*We previously interrogated the significance of two other purported RNA binding regions of CTCF, ZF1 and ZF10 (Hyle 2023). Our data showed loss of these regions lead to unique differential binding patterns specific to each ZF that could be correlated to differential gene expression of a subset of genes regulated by CTCF. Motif analysis also identified each ZF preferentially bound to DNA at a characteristic motif that included the CTCF core consensus motif along with an upstream or downstream motif signature unique to each ZF. This data suggested these ZFs bound directly to the DNA to regulate gene expression. To date, the RBR, ZF1 and ZF10 are the only purported regions of CTCF to bind RNA that we have studied. However, other regions of the protein could serve this purpose. In the future, we plan to comprehensively screen for additional essential RNA-binding regions. Using our AID system and inducible expression platform, we will systematically investigate the function of potential RNA-binding regions by integrative ChIP-seq and RNA-seq analysis.*

*Last but not the least, we appreciate the suggestion to use Diffbind algorithm to re-run our ChIP-seq data. For the differential peak identification method used in our study, we estimated the normalization factors using the TMM method, and the counts were normalized using the voom function. The lmFit and eBayes functions, with default parameters from the limma R package, were then applied to the voom-normalized counts to identify differential peaks. During the revision, we used the suggested DiffBind to identify differential peaks between dRBR and WT CTCF through the DiffBind R package (version 3.10.1). Briefly, for each comparison pair, consensus sites were identified and extended 250 base pairs upstream and downstream from the summit of each interval, and read counts were calculated. Differential peak analysis was then performed using the default DESeq2 function. Fold change values were then calculated based on the DiffBind normalized counts. Differential peaks were identified using four different cutoff stringency with FDR or P < 0.05 and FC > 1 or 2 (shown below, Data 1 for the reviewer). Again, the data are consistent to our previous observation with even higher stringency. Thank you for the great suggestion to further consolidate our conclusion. We will consider including the DiffBind algorithm in all our future analysis as a complementary approach.*

**Data 1 for the reviewer**

2. Gene expression data shown in Data 5 for reviewer only have 2 replicates. This is inconsistent with the authors' claim that the experiments were done in 3 replicates. The authors should clarify. 3 replicates for gene expression profiling should be the norm in the field.

*Response: Thank you for this comment. The RNA-seq comparison between CTCF-WT and CTCF-dRBR has been repeated to include three replicates of each. CTCF^AID2/WT^ and CTCF^AID2/dRBR^ cells were treated in triplicate to degrade the endogenous CTCF^miniAIDmClover^ protein and exogenously express doxycycline-inducible HA-tagged CTCF-WT or CTCF-dRBR. Protein expression was consistent between replicates and cell lines. A non-treated control (NoT) was included for the immunoblot (Data 2 for the reviewer). No differential gene expression was observed at FDR < 0.05. The data have been updated in Fig. 4E, 4F, and Figure S4D-S4F. Please see the original data and new data below.*

**Data 2 for the reviewer**

**Original Fig. 4E**

**Now Figure S4E**

**New Fig. 4E**

**Original Fig. 4F**

**New Fig. 4F**

**New Figure S4F**

**Original S4D**

**New Figure S4D**

3. Data 6 for the reviewer only shows the CTCF binding patterns in a narrow window (10kb-50kb) around the gene. As CTCF can induce loops and 3D chromatin changes in a larger scale, the authors should examine at least 100-200kb regions.

*Response: This comment is regarding ChIP-seq tracks for three genes identified by RNA-seq to be differentially expressed (FDR < 0.05) when comparing gene expression between cells expressing either WT or dRBR CTCF (N=2). When the RNA-seq was repeated with three replicates, the differentially expressed genes identified from the original RNA-seq no longer showed differential expression at FDR < 0.05. For your reference, the ChIP-seq tracks for these loci have been expanded. As expected, no differential CTCF binding was observed in the larger window (Data 3-5 for the reviewer).*

**Data 3 for the reviewer**

**Data 4 for the reviewer**

**Data 5 for the reviewer**

**Second round of review**

**Reviewer 3**

I commend the authors for thoroughly addressing all my comments on the previous version.
